# Stepwise, Protecting Group Free Synthesis of [4]Rotaxanes

**DOI:** 10.3390/molecules22010089

**Published:** 2017-01-09

**Authors:** James E. M. Lewis, Joby Winn, Stephen M. Goldup

**Affiliations:** 1 Chemistry, University of Southampton, Highfield, Southampton SO17 1BJ, UK; jem.lewis@soton.ac.uk; 2 School of Biological and Chemical Sciences, Queen Mary University of London, Mile End Road, London E1 4NS, UK; joby.winn@googlemail.com

**Keywords:** rotaxane, mechanically interlocked, copper-catalyzed azide-alkyne cycloaddition, CuAAC, active template

## Abstract

Despite significant advances in the last three decades towards high yielding syntheses of rotaxanes, the preparation of systems constructed from more than two components remains a challenge. Herein we build upon our previous report of an active template copper-catalyzed azide-alkyne cycloaddition (CuAAC) rotaxane synthesis with a diyne in which, following the formation of the first mechanical bond, the steric bulk of the macrocycle tempers the reactivity of the second alkyne unit. We have now extended this approach to the use of 1,3,5-triethynylbenzene in order to successively prepare [2]-, [3]- and [4]rotaxanes without the need for protecting group chemistry. Whilst the first two iterations proceeded in good yield, the steric shielding that affords this selectivity also significantly reduces the efficacy of the active template (AT)-CuAAC reaction of the third alkyne towards the preparation of [4]rotaxanes, resulting in severely diminished yields.

## 1. Introduction

Although the first synthesis of a rotaxane was reported in 1967 [1], the development of convenient and high yielding methods for preparing mechanically interlocked molecules (MIMs) remains an active area of research due to their potential applications [2,3,4,5,6] in fields as diverse as sensing [7,8,9], catalysis [10,11,12], medicine [13,14,15,16,17,18] and as artificial molecular machines [19,20,21]. Following Sauvage’s ground-breaking use of metal ions as templating species for the synthesis of MIMs [22,23], a variety of interactions have been utilized to arrange components prior to formation of the final covalent bond that establishes the mechanical bond [24,25]. These have included coordination bonds with a range of main group and transition row metal ions [26,27,28], anion-templation [29,30,31,32], π-stacking interactions [33], H-bonding [34,35] and radical-radical interactions [36], amongst others. However, the synthesis of more complex rotaxanes in which two or more macrocycles encircle a thread remains challenging [37,38,39,40,41,42,43,44,45].

Whilst the use of non-covalent interactions to direct the self-assembly of oligo[n]pseudorotaxanes prior to formation of a permanent mechanical bond is the most operationally simple method towards forming multi-component interlocked species [46,47,48,49,50,51], this generally offers little in the way of control over structural or sequence specificity. A limited number of examples of self-sorting systems have been reported [49,50,51,52,53,54]. However, iterative approaches [55,56,57,58,59,60,61] tend to offer the best opportunity for rapid and economical formation of precision designed oligo[n]rotaxanes.

**Scheme 1:**
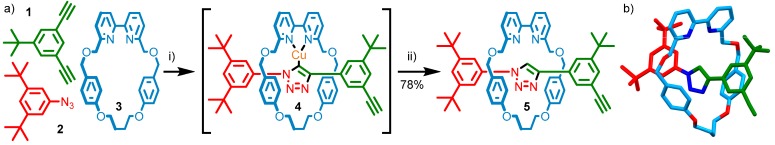
(**a**) Synthesis of [2]rotaxane **5** from diyne **1**. *Reagents and conditions*: (i) 1 equiv. each of **1**, **2**, **3**, [Cu(CH_3_CN)_4_](PF_6_) and *i*-Pr_2_NEt, EtOH, 100 °C (microwave, μw), 2 h; (ii) CH_2_Cl_2_, 100 °C (μw), 1 h; (**b**) X-ray crystal structure of **5**.

We have developed a *small macrocycle* [62] modification of Leigh’s active template Cu-mediated azide-alkyne cycloaddition (AT-CuAAC) methodology [63] that enables the preparation of rotaxanes in very high yield, and removes the need for extraordinarily large stoppering units [64]. As part of a preliminary investigation into the synthesis of oligo[n]rotaxanes through an iterative methodology, we recently reported that the steric crowding of the macrocycle around functional stoppering substituents modulates their reactivity; in this manner a diyne (**1**) could be reacted under AT-CuAAC conditions to give exclusive formation of a [2]rotaxane, rather than the anticipated statistical mixture of [2]- and [3]rotaxanes, due to the formation of a stable Cu(I)-triazolide intermediate [65] that hindered access to the second alkyne moiety (Scheme 1). Following purification, the [2]rotaxane could be subjected to another iteration of the reaction conditions, resulting in formation of the [3]rotaxane architecture [66].

With a view to extending this approach towards more complex dendritic-type systems [61,67,68], we herein report the use of a triyne (**6**) in a similar manner, allowing the stepwise, high-yielding synthesis of [2]- and [3]rotaxanes. Disappointingly, due to the steric crowding which allows us to conduct this protecting-group free synthesis, the attempted formation of [4]rotaxanes proceeded in severely diminished yields. Through the use of larger, more flexible macrocycles and less sterically encumbered stoppering units the yield could be increased, but failed to give the triply-interlocked species as the major product.

## 2. Results and Discussion

### 2.1. Stepwise Synthesis of [2]- and [3]Rotaxanes

**Scheme 2:**
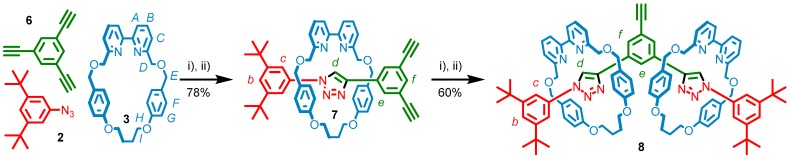
Stepwise synthesis of [2]rotaxane **7**, and [3]rotaxane **8**. *Reagents and conditions*: (i) 1 equiv. each of **6**, **2**, **3**, [Cu(CH_3_CN)_4_](PF_6_) and *i*-Pr_2_NEt, EtOH, 100 °C (μW), 2 h; (ii) CH_2_Cl_2_, 100 °C (μW), 1 h.

1,3,5-Triethynylbenzene, (**6**) was subjected to the optimized reaction conditions previously established for diyne **1** with the intention of exclusively forming [2]rotaxane, **7** (Scheme 2). As such **6** was reacted with one equivalent each of macrocycle **3**, azide **2**, [Cu(CH_3_CN)_4_](PF_6_) and *i*-Pr_2_NEt in EtOH at 100 °C under microwave irradiation for 2 h, followed by a solvent swap to CH_2_Cl_2_ and an additional hour heating at 100 °C to effect protonolysis of the Cu-triazolide intermediate [69]. Pleasingly, analysis of the ^1^H-NMR spectrum of the crude reaction mixture revealed complete consumption of the starting materials, and almost exclusive formation of the desired [2]rotaxane. After purification by column chromatography, compound **7** was obtained in 78% isolated yield, and its identity confirmed by NMR (*vide infra*) and mass spectrometry (MS, *m/z* = 864.45 [M + H]^+^).

With **7** in hand the next step was to examine whether [3]rotaxane **8** could be obtained under the same conditions (Scheme 2). Pleasingly, submission of **7** to another iteration of the reaction conditions with a further equivalent each of **2**, **3**, [Cu(CH_3_CN)_4_](PF_6_) and *i*-Pr_2_NEt gave, following column chromatography, **8** (*m/z* = 1577.87 [M + H]^+^) in 60% isolated yield.

### 2.2. Analysis of [2]- and [3]Rotaxanes ***7*** and ***8***

**Figure 1:**
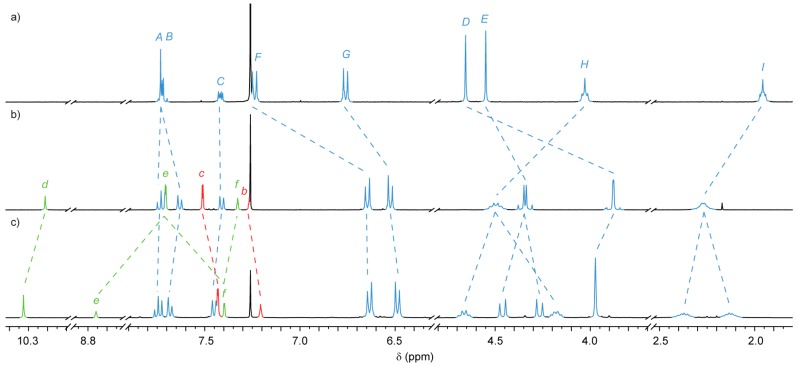
^1^H-NMR spectra (400 MHz, CDCl_3_, 298 K) of (**a**) macrocycle **3**; (**b**) [2]rotaxane **7**; and (**c**) [3]rotaxane **8**. For macrocycle and thread labeling see Scheme 2.

A comparison of the ^1^H-NMR spectra of **7** and **8** (Figure 1b,c, respectively) shows that, as expected, the overall symmetry of the thread does not change following the second AT-CuAAC reaction; however, some peaks were found to resonate at different chemical shifts. The triazole proton (H*_d_*) shifts 0.12 ppm further downfield, whilst those of the terminal stoppering units (H*_b_* and H*_c_*) were observed further upfield (0.06 and 0.09 ppm, respectively).

Most interestingly the diastereotopic protons of the methylene units of the macrocycle butylene chain of **8** appear as distinct multiplets (Δδ = 0.48 and 0.23 ppm for H*_H_* and H*_I_*, respectively). This is presumably a result of the increased steric hindrance from the presence of two macrocycles in close proximity, reducing flexibility of the butylene chain. H*_E_*, the methylene unit adjacent to the flanking aromatic moieties of the macrocycle, was also observed to split into two distinct doublets.

**Figure 2:**
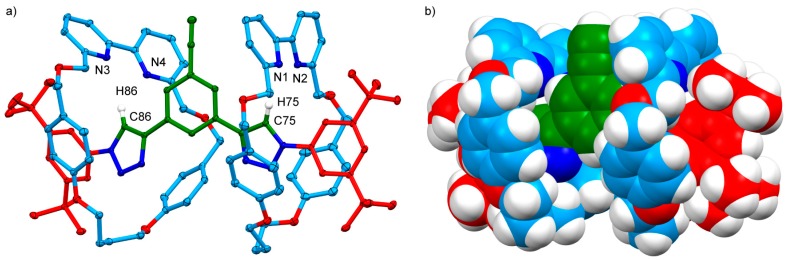
X-ray crystal structure of **8** shown as (**a**) ellipsoid; and (**b**) space-filling plots. Ellipsoids are shown at 50% probability. Some hydrogen atoms have been omitted for clarity. Interactions lengths (Å) and distances (°): H75···N1 2.392, H75···N2 2.688, C75-H75···N1 167.3, C75-H75···N2 126.0, H86···N3 2.541, H86···N4 2.534, C86-H86···N3 156.3, C86-H86···N4 135.6.

As we have previously found with similar [3]rotaxane structures [66], proton H*_e_*—sandwiched between the two triazole moieties on the thread—was found to resonate 1.05 ppm higher than the corresponding proton (H*_e_*) in the [2]rotaxane, indicative of a *syn*-*syn* arrangement of the two triazole rings. X-ray quality crystals of **8** were obtained by vapor diffusion of pentane into a CH_2_Cl_2_ solution, allowing the solid state structure to be determined by crystallography (Figure 2). The structure shows the expected hydrogen-bond interactions between the protons of the triazole rings and the bipyridine moieties of the macrocycles (C-H···N distance 2.4–2.7 Å), and reveals the thread to be in the *syn-syn* conformation suggested by the solution phase ^1^H-NMR data.

### 2.3. Synthesis of [4]Rotaxanes

**Scheme 3:**
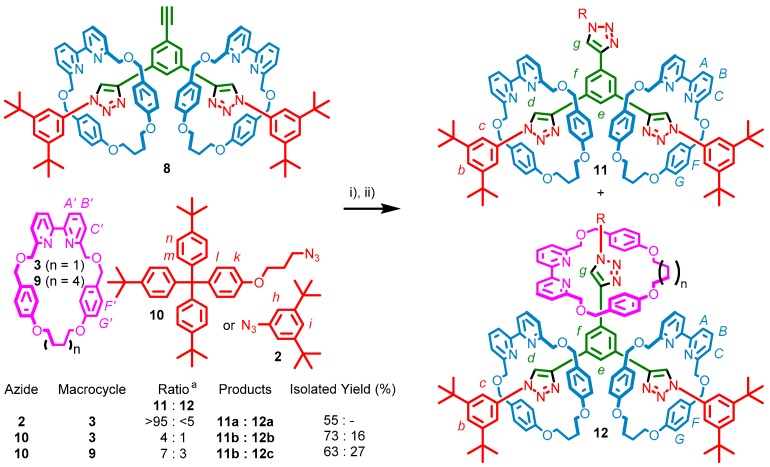
Synthesis of [4]rotaxanes. *Reagents and conditions*: (i) 1 equiv. each of **2** or **10**, **3** or **9**, [Cu(CH_3_CN)_4_](PF_6_) and *i*-Pr_2_NEt, EtOH, 100 °C (μW), 2 h; (ii) CH_2_Cl_2_, 100 °C (μW), 1 h. [a] Determined by ^1^H-NMR.

Having successfully prepared **8** in two steps in good yield (47% overall), a third AT-CuAAC reaction was attempted to prepare the desired [4]rotaxane, **12a** (Scheme 3). Submission of **8** to another iteration of the reaction conditions resulted in a crude NMR which appeared to show a single species, with peaks shifted relative to **8**. However, following column chromatography, macrocycle **3** was recovered, along with a new product, identified as the tris-triazole thread encircled by two macrocycles (**11a**; *m/z* = 905.01 [M + 2H]^2+^).

**Figure 3:**
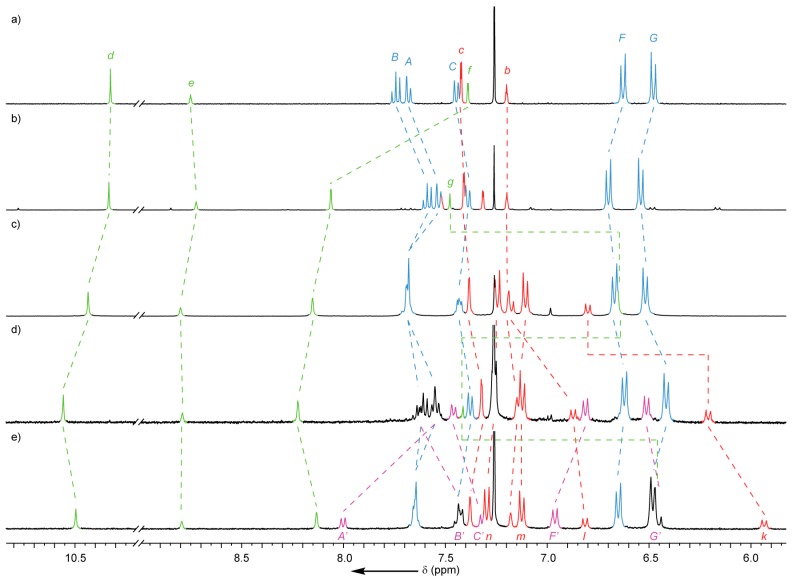
Partial ^1^H-NMR spectra (CDCl_3_, 400 MHz, 298 K) of [3]rotaxanes (**a**) **8**; (**b**) **11a**; (**c**) **11b**; and [4]rotaxaes (**d**) **12b** and (**e**) **12c**. For macrocycle and thread labeling see Scheme 3.

Closer inspection of the ^1^H-NMR spectrum of this purified [3]rotaxane (Figure 3b) revealed a small amount (<5%) of impurity to be present that had co-eluted with the major product (re-examination of the crude reaction mixture showed a similar ratio of this impurity). Although a weak signal consistent with the desired [4]rotaxane could be observed by MS (*m/z* = 1146 [M + 2H]^2+^; see Supplementary Materials), the identity of this species could not be reliably confirmed, and as such it could not be determined conclusively whether any **12a** had formed.

It was assumed that the steric bulk of the two existing macrocycles was hindering access to the third alkyne moiety. To alleviate some of this crowding, the reaction was repeated using azide **10** which incorporated an alkyl chain. The crude ^1^H-NMR spectrum of the reaction mixture revealed two products, with the ratio of these estimated at 1:4 based on the integration values of two downfield-shifted triazole proton resonances at 10.56 and 10.44 ppm, respectively (see Supplementary Materials). Pleasingly, the products were able to be separated by column chromatography. The major species, isolated in 73% yield, was identified as the product of the reaction between **8** and **10**—without trapping of macrocycle **3**—by the presence of resonances consistent with the new stoppering unit, but lacking any signals attributable to a third macrocycle (Figure 3c), with MS data supporting this conclusion (*m/z* = 1082.13 [M + 2H]^2+^).

The minor species, isolated in 16% yield, showed additional peaks in the ^1^H-NMR spectrum consistent with a mechanically trapped macrocycle in a different environment to the pre-existing two (Figure 3d, *vide infra*). With additional evidence from MS (*m/z* = 1324.23 [M + 2H]^2+^), this species was assigned as the [4]rotaxane product, **12b**.

Subsequently, in a further attempt to decrease steric crowding and increase the yield of the [4]rotaxane product, the effect of increasing the size of the macrocycle was investigated by employing the larger macrocycle **9**. The reaction between **8**, azide **10** and macrocycle **9** again yielded two products, this time in a ratio of 3:7, as judged by ^1^H-NMR analysis of the crude reaction mixture (see Supplementary Materials). The ^1^H-NMR spectrum of the major species was identical to the previously isolated [3]rotaxane, **11b**, whilst the minor species again exhibited additional signals consistent with the presence of a third macrocycle in a different chemical environment to the other two (Figure 3e). Following column chromatography the major species was isolated in 63% yield, and its identity confirmed as the [3]rotaxane **11b**. Isolated in 27% yield, the minor species was identified as the anticipated [4]rotaxane, **12c**, by NMR (*vide infra*) and MS (*m/z* = 1366.27 [M + 2H]^2+^). As such, through alleviation of the steric crowding around the incoming macrocycle and azide, the yield of the third AT-CuAAC reaction was able to be increased from <5% to a respectable 27%.

### 2.4. Analysis of [4]Rotaxanes ***12b*** and ***12c***

The ^1^H-NMR spectra of the [3]rotaxanes (**11a** and **11b**) and [4]rotaxanes (**12b** and **12c**) each display characteristic peaks related to their structure and conformation in solution (Figure 3). The resonance for the proton *ortho* to the two equivalent triazole rings (H*_e_*) does not shift significantly in any of these species, suggesting that the rotaxane maintains the *syn*-*syn* conformation observed in solution and the solid state for [3]rotaxane **8**. The signal for the phenyl protons adjacent to the third alkyne (H*_f_*) was found to shift downfield by approximately 0.7 ppm (Δδ = 0.66 − 0.82) upon formation of the third triazole ring in the [3]- and [4]rotaxanes, presumably similarly due to interactions between the protons and the triazole ring; the observation of a single signal suggests free rotation of the phenyl-triazole bond on the NMR timescale.

The ^1^H-NMR spectra of both [4]rotaxanes display some noticeable shifts relative to the corresponding [3]rotaxane, **11b**, consistent with formation of an interlocked molecule. The aromatic peaks of the trityl stoppering unit closest to the triazole, H*_k_* and H*_l_*, shift significantly upfield in the presence of macrocycles **3** (Δδ = 0.59 and 0.31, respectively) and **9** (Δδ = 0.86 and 0.36, respectively) in **12b** and **12c**, respectively. Crucially the resonances of the third macrocycle were shifted relative to the free component; particularly those associated with the flanking aromatic units of the macrocycles (H*_F’_* and H*_G’_*) were found to shift downfield relative to the free macrocycle, consistent with an interlocked structure.

As previously reported [64], the size of the third macrocycle greatly affects the hydrogen bonding interaction between the bipyridine unit and the triazole proton of the thread (H*_g_*), as evidenced by the chemical shift of the latter. In the case of [4]rotaxane **12c** with the larger macrocycle **9**, the triazole proton is observed at a similar chemical shift to the non-interlocked component of the corresponding [3]rotaxane **11b** (6.44 vs. 6.65 ppm, respectively). Conversely, in the [4]rotaxane **12b** with the smaller macrocycle **3**, H*_g_* resonates further downfield at 7.41 ppm.

## 3. Materials and Methods

### 3.1. General Information

Unless otherwise stated, all reagents, including anhydrous solvents, were purchased from commercial sources and used without further purification. All reactions were carried out under an atmosphere of N_2_ using anhydrous solvents unless otherwise stated. Petrol refers to the fraction of petroleum ether boiling in the range 40–60 °C. EDTA-NH_3_ solution refers to an aqueous solution of NH_3_ (17% *w*/*w*) saturated with sodium-ethylenediaminetetraacetate. Flash column chromatography was performed using Biotage Isolera-4 automated chromatography system, employing Biotage SNAP or ZIP cartridges. Analytical TLC was performed on precoated silica gel plates (0.25 mm thick, 60F254, Merck, Darmstadt, Germany) and observed under UV light. Microwave reactions were completed using a CEM Discover S Microwave system. Reactions were run at a power level of 150 W.

NMR spectra were recorded on Bruker AV400, AV3-400, AV500 or Bruker AV600 instrument, at a constant temperature of 298 K. Chemical shifts are reported in parts per million from low to high field and referenced to residual solvent. Coupling constants (*J*) are reported in Hertz (Hz). Standard abbreviations indicating multiplicity were used as follows: m = multiplet, quint = quintet, q = quartet, t = triplet, d = doublet, s = singlet, app. = apparent, br = broad. Signal assignment was carried out using 2D NMR methods (HSQC, HMBC, COSY, NOESY) where necessary. In the case of some complex multiplets with contributions from more than one signal absolute assignment was not possible. Here indicative either/or assignments (e.g., H*_A/B_* for H*_A_* or H*_B_*) are provided. All melting points were determined using a Griffin apparatus. Low resolution mass spectrometry was carried out by the mass spectrometry services at the University of Southampton (Waters TQD mass spectrometer equipped with a triple quadrupole analyser with UHPLC injection [BEH C18 column; MeCN-hexane gradient {0.2% formic acid}]). High resolution mass spectrometry was carried out by the mass spectrometry services at the University of Southampton (MaXis, Bruker Daltonics, with a Time of Flight (TOF) analyser; samples were introduced to the mass spectrometer via a Dionex Ultimate 3000 autosampler and uHPLC pump in a gradient of 20% acetonitrile in hexane to 100% acetonitrile (0.2% formic acid) over 5 min at 0.6 mL min; column: Acquity UPLC BEH C18 (Waters) 1.7 micron 50 × 2.1mm).

The following compounds were synthesized according to literature procedures: 1-azido-3,5-di-*tert*-butylbenzene (**2**) [70], 1,3,5-triethynylbenzene (**6**) [71], azide stopper **10** [63], and macrocycles **3** and **9** [62].

### 3.2. General Procedure

*i-*Pr_2_NEt (0.1 M in EtOH, 1 eq.) was added to a solution of macrocycle (1 eq.), [Cu(CH_3_CN)_4_]PF_6_ (1 eq.), alkyne (1 eq.) and azide (1 eq.) in EtOH (2.5 mL) in a CEM vial. The deep red reaction mixture was stirred at 100 °C under microwave irradiation for 2 h. After removal of the solvent in vacuo the residue was dissolved in CH_2_Cl_2_ (2.5 mL) and stirred at 100 °C under microwave irradiation for 1 h. To the cooled reaction mixture was added EDTA-NH_3_ solution (50 mL) and extracted with CH_2_Cl_2_ (3 × 50 mL). The combined organic extracts were dried (MgSO_4_) and the solvent removed in vacuo. Purification by flash column chromatography on silica gel yielded the product.

#### 3.2.1. Synthesis of [2]Rotaxane **7**

General procedure. **6** (22.5 mg, 0.15 mmol, 1 eq.), **2** (34.7 mg, 0.15 mmol, 1 eq.), **3** (72.3 mg, 0.15 mmol, 1 eq.), [Cu(CH_3_CN)_4_](PF_6_) (55.9 mg, 0.15 mmol, 1 eq.). Purification by column chromatography on silica gel (petrol with 0% to 100% gradient of Et_2_O) yielded the product as a white solid (101 mg, 78%). m.p. = 171–174 °C. ^1^H-NMR (600 MHz, CDCl_3_) δ: 10.22 (s, 1H, H*_d_*), 7.74–7.71 (m, 4H, H*_B_*, H*_e_*), 7.63 (d, *J* = 7.8, 2H, H*_A_*), 7.52 (d, *J* = 1.6, 2H, H*_c_*), 7.41 (d, *J* = 7.7, 2H, H*_C_*), 7.33 (t, *J* = 1.4, 1H, H*_f_*), 7.27 (t, *J* = 1.6, 1H, H*_b_*), 6.65 (d, *J* = 8.5, 4H, H*_F_*), 6.53 (d, *J* = 8.5, 4H, H*_G_*), 4.52–4.48 (m, 4H, H*_H_*), 4.37–4.32 (m, 4H, H*_E_*), 3.91–3.86 (m, 4H, H*_D_*), 2.93 (s, 2H, H*_g_*), 2.29–2.25 (m, 4H, H*_I_*), 1.21 (s, 18H, H*_a_*). ^13^C-NMR (151 MHz, CDCl_3_) δ: 159.4, 159.2, 155.9, 151.8, 144.0, 137.4, 137.1, 133.6, 132.1, 130.0, 129.1, 128.0, 121.9, 121.0, 120.3, 115.2, 114.6, 83.1, 77.2 (via HSQC), 73.1, 70.4, 66.5, 35.2, 31.4, 24.9. ESI-MS *m/z* = 864.4484 [M + H]^+^ calc. 864.4483.

#### 3.2.2. Synthesis of [3]Rotaxane **8**

General procedure. **7** (60.4 mg, 0.069 mmol, 1 eq.), **2** (16.0 mg, 0.069 mmol, 1 eq.), **3** (33.3 mg, 0.069 mmol, 1 eq.), [Cu(CH_3_CN)_4_](PF_6_) (25.3 mg, 0.069 mmol, 1 eq.). Purification by column chromatography on silica gel (petrol with 0% to 100% gradient of Et_2_O) yielded the product as a white solid (65 mg, 60%). m.p. = 226–228 °C. ^1^H-NMR (400 MHz, CDCl_3_) δ: 10.33 (s, 2H, H*_d_*), 8.76 (s, 1H, H*_e_*), 7.74 (t, *J* = 7.7, 4H, H*_B_*), 7.68 (d, *J* = 7.4, 4H, H*_A_*), 7.45 (d, *J* = 7.6, 4H, H*_C_*), 7.43 (d, *J* = 1.6, 4H, H*_c_*), 7.40 (d, *J* = 1.5, 2H, H*_f_*), 7.21 (t, *J* = 1.5, 2H, H*_b_*), 6.63 (d, *J* = 8.5, 8H, H*_F_*), 6.49 (d, *J* = 8.5, 8H, H*_G_*), 4.66 (app. q, *J* = 7.4, 4H, H*_H_*), 4.46 (d, *J* = 12.2, 4H, 4 of H*_E_*), 4.27 (d, *J* = 12.2, 4H, 4 of H*_E_*), 4.21–4.15 (m, 4H, 4 of H*_H_*), 3.97 (s, 8H, H*_D_*), 2.61 (s, 1H, H*_g_*), 2.40–2.33 (m, 4H, 4 of H*_I_*), 2.17–2.09 (m, 4H, 4 of H*_I_*), 1.19 (s, 36H, H*_a_*). ^13^C-NMR (101 MHz, CDCl_3_) δ: 159.7, 159.3, 155.6, 151.4, 145.1, 137.4, 137.2, 132.1, 129.0, 128.5, 127.9, 121.0, 120.7, 120.2, 115.2, 114.8, 84.2, 75.6, 73.1, 70.4, 66.5, 35.1, 31.5, 25.1. ESI-MS *m/z* = 1577.87 [M + H]^+^ calc. 1577.84; 789.44 [M + 2H]^2+^ calc. 789.42.

#### 3.2.3. Synthesis of [3]Rotaxane **11a**

General procedure. **8** (16.1 mg), **2** (4.9 mg, 0.010 mmol, 1 eq.), **3** (4.9 mg, 0.010 mmol, 1 eq.), [Cu(CH_3_CN)_4_](PF_6_) (3.7 mg, 0.010 mmol, 1 eq.). Purification by column chromatography on silica gel (petrol with 0% to 100% gradient of Et_2_O) yielded the **11a** as a white foam (10.2 mg, 55%). ^1^H-NMR (CDCl_3_, 400 MHz, 298 K) δ: 10.33 (s, 2H, H*_d_*), 8.72 (br. m, 1H, H*_e_*), 8.06 (d, *J* = 1.5, 2H, H*_f_*), 7.59 (app. t, *J* = 7.7, 4H, H*_B_*), 7.54–7.51 (m, 5H, H*_A_*, H*_i_*), 7.48 (s, 1H, H*_g_*), 7.41 (d, *J* = 1.6, 4H, H*_c_*), 7.39 (d, *J* = 7.5, 4H, H*_C_*), 7.32 (d, *J* = 1.7, 2H, H*_h_*), 7.20 (t, *J* = 1.5, 2H, H*_b_*), 6.70 (d, *J* = 8.5, 8H, H*_F_*), 6.54 (d, *J* = 8.5, 8H, H*_G_*), 4.79–4.73 (m, 4H, 4 of H*_H_*), 4.50 (d, *J* = 12.2, 4H, 4 of H*_E_*), 4.28 (d, *J* = 12.1, 4H, 4 of H*_E_*), 4.20–4.15 (m, 4H, 4 of H*_H_*), 4.07–4.00 (m, 8H, H*_D_*), 2.43 (br. m, 4H, 4 of H*_I_*), 2.12 (br. m, 4H, 4 of H*_I_*), 1.41 (s, 18H, H*_j_*), 1.18 (s, 36H, H*_a_*). ^13^C-NMR (CDCl_3_, 101 MHz, 298 K) δ: 159.8, 159.4, 155.5, 152.6, 151.3, 148.7, 145.8, 137.4, 137.2, 137.2, 132.6, 130.3, 129.2, 127.9, 122.9, 122.7, 122.3, 122.2, 121.1, 120.6, 120.2, 118.5 (via HSQC), 115.9, 115.2, 114.8, 73.2, 70.4, 66.6, 35.3, 35.1, 31.6, 31.5, 25.1. ESI-MS *m/z* = 905.01 [M + 2H]^2+^ calc. 905.01.

#### 3.2.4. Synthesis of [3]Rotaxane **11b** and [4]Rotaxane **12b**

General procedure. **8** (17.5 mg, 0.011 mmol, 1 eq.), **10** (6.5 mg, 0.011 mmol, 1 eq.), **3** (5.4 mg, 0.011 mmol, 1 eq.), [Cu(CH_3_CN)_4_](PF_6_) (4.0 mg, 0.011 mmol, 1 eq.). Purification by column chromatography on silica gel (petrol with 0 to 100% gradient of Et_2_O) yielded **11b** (17.5 mg, 73%) and **12b** (4.7 mg, 16%) as white foams.

[3]Rotaxane **11b**. ^1^H-NMR (CDCl_3_, 400 MHz, 298 K) δ: 10.44 (s, 2H, H*_d_*), 8.80 (s, 1H, H*_e_*), 8.15 (d, *J* = 0.8, 2H, H*_f_*), 7.71–7.68 (m, 8H, H*_A_*, H*_B_*), 7.44–7.42 (m, 4H, H*_C_*), 7.38 (d, *J* = 1.3, 4H, H*_c_*), 7.24 (d, *J* = 8.5, 6H, H*_m_* or H*_n_*), 7.19–7.17 (m, 3H, H*_b_*, H*_l_*), 7.11 (d, *J* = 8.5, 6H, H*_m_* or H*_n_*), 6.80 (d, *J* = 8.8, 2H, H*_k_*), 6.68–6.65 (m, 9H, H*_F_*, H*_g_*), 6.52 (d, *J* = 8.5, 8H, H*_G_*), 4.79 (app. q, *J* = 7.6, 4H, 4 of H*_H_*), 4.53 (d, *J* = 12.2, 4H, 4 of H*_E_*), 4.22–4.12 (m, 10H, 4 of H*_E_*, 4 of H*_H_*, H*_h_*), 4.05–3.98 (m, 8H, H*_D_*), 3.87 (t, *J* = 5.5, 2H, H*_j_*), 2.45 (br. m, 4H, 4 of H*_I_*), 2.10 (br. m, 6H, 4 of H*_I_*, H*_i_*), 1.30 (s, 27H, H*_o_*), 1.17 (s, 36H, H*_a_*). ^13^C-NMR (CDCl_3_, 101 MHz, 298 K) δ: 160.1, 159.4, 156.5, 155.3, 151.3, 148.6, 148.5, 145.8, 144.2, 140.5, 137.4, 137.4, 132.7, 132.6, 130.9, 130.4, 129.2, 127.8, 125.7, 124.3, 122.4, 122.1, 122.0, 121.2, 120.5, 120.0, 115.1, 114.7, 113.1, 73.2, 70.3, 66.5, 64.3, 63.2, 47.0, 35.1, 34.5, 31.5, 31.5, 30.3, 25.1. ESI-MS *m/z* = 1083.12 [M + 2H]^2+^ calc. 1083.12.

[4]Rotaxane **12b**. ^1^H-NMR (CDCl_3_, 500 MHz, 298 K) δ: 10.56 (s, 2H, H*_d_*), 8.79 (s, 1H, H*_e_*), 8.22 (br. m, 2H, H*_f_*), 7.65–7.59 (m, 6H, H*_B_*, H*_B’_*), 7.56–7.53 (m, 6H, H*_A_*, H*_A’_*), 7.47–7.56 (m, 2H, H*_C’_*), 7.41 (s, 1H, H*_g_*), 7.38 (d, *J* = 7.6, 4H, H*_C_*), 7.32 (d, *J* = 1.7, 4H, H*_c_*), 7.27–7.25 (m, 6H, H*_n_*), 7.15 (s, 2H, H*_b_*), 7.12 (d, *J* = 8.6, 6H, H*_m_*), 6.87 (d, *J* = 8.6, 2H, H*_k/l_*), 6.81 (d, *J* = 8.3, 4H, H*_F’_*), 6.62 (d, *J* = 8.3, 8H, H*_F_*), 6.51 (d, *J* = 8.4, 4H, H*_G’_*), 6.42 (d, *J* = 8.3, 8H, H*_G_*), 6.21 (d, *J* = 8.6, 2H, H*_k/l_*), 4.71–4.67 (m, 4H, 4 of H*_H_*), 4.60–4.52 (m, 6H, 4 of H*_E_*, 2 of H*_E’_*), 4.35–4.32 (m, 4H, 2 of H*_D’_*, 2 of H*_E’_*), 4.23 (d, *J* = 12.4, 2H, 2 of H*_D’_*), 4.17–4.14 (6H, 4 of H*_E_*, 2 of H*_H’_*), 4.09 (d, *J* = 13.3, 4H, 4 of H*_D_*), 4.00 (d, *J* = 13.3, 4H, 4 of H*_D_*), 3.93 (br. m, 6H, 4 of H*_H_*, 2 of H*_H’_*), 3.30–3.25 (m, 2H, H*_h/j_*), 2.89 (t, *J* = 6.9, 2H, H*_h/j_*), 2.37 (br. m, 4H, 4 of H*_I_*), 2.07–1.92 (m, 8H, 4 of H*_I_*, H*_I’_*), 1.32 (s, 27H, H*_o_*), 1.13 (s, 36H, H*_a_*), 0.84 (br. m, 2H, H*_i_*). ^13^C-NMR (CDCl_3_, 126 MHz, 298 K) δ: 159.8, 159.0, 158.9, 156.6, 156.3, 155.3, 151.0, 148.2, 147.1, 145.7, 144.4, 138.6, 137.2, 137.1 (×2), 135.2, 132.8, 131.6, 131.3, 130.7, 130.2, 128.9, 128.8, 127.7, 124.0, 122.4, 122.1, 122.0, 121.1, 120.7, 120.5, 120.3, 119.9 (×2), 115.2, 114.9, 114.6, 113.1, 72.9, 72.8, 70.7, 70.2, 66.7, 66.1, 64.0, 63.1, 46.3, 34.9, 34.3, 31.4, 31.3, 29.0, 24.9, 24.8. ESI-MS *m/z* = 883.16 [M + 3H]^3+^ calc. 883.16; 1324.23 [M + 2H]^2+^ calc. 1324.23.

#### 3.2.5. Synthesis of [3]Rotaxane **11b** and [4]Rotaxane **12c**

General procedure. **8** (20.5 mg, 0.013 mmol, 1 eq.), **10** (7.6 mg, 0.013 mmol, 1 eq.), **9** (7.4 mg, 0.013 mmol, 1 eq.), [Cu(CH_3_CN)_4_](PF_6_) (4.6 mg, 0.013 mmol, 1 eq.). Purification by column chromatography on silica gel (petrol with 0 to 100% gradient of Et_2_O) yielded **11b** (17.9 mg, 63%) and **12c** (9.7 mg, 27%) as white foams.

[4]Rotaxane **12c**. ^1^H-NMR (CDCl_3_, 500 MHz, 298 K) δ: 10.50 (s, 2H, H*_d_*), 8.79 (s, 1H, H*_e_*), 8.13 (s, 2H, H*_f_*), 8.00 (d, *J* = 7.7, 2H, H*_A’_*), 7.68–7.65 (m, 8H, H*_A_*, H*_B_*), 7.45–7.42 (m, 6H, H*_C_*, H*_B’_*), 7.38 (s, 4H, H*_c_*), 7.32 (d, *J* = 7.9, 2H, H*_C’_*), 7.30 (d, *J* = 8.7, 6H, H*_m/n_*), 7.18 (s, 2H, H*_b_*), 7.13 (d, *J* = 8.7, 6H, H*_m/n_*), 6.96 (d, *J* = 8.6, 4H, H*_F’_*), 6.82 (d, *J* = 8.8, 2H, H*_l_*), 6.65 (d, *J* = 8.5, 8H, H*_F_*), 6.49–6.44 (m, 13H, H*_G_*, H*_G’_*, H*_g_*), 5.94 (d, *J* = 8.7, 2H, H*_k_*), 4.76 (br. m, 4H, 4 of H*_H_*), 4.64–4.53 (m, 12H, 4 of H*_E_*, H*_D’_*, H*_E’_*), 4.19 (d, *J* = 12.2, 4H, H*_E_*), 4.08 (br. m, 4H, 4 of H*_H_*), 4.04 (d, *J* = 13.3, 4H, 4 of H*_D_*), 4.00 (d, *J* = 13.2, 4H, 4 of H*_D_*), 3.73–3.67 (m, 4H, H*_H’_*), 3.61 (br. m, 2H, H*_h/j_*), 2.94 (br. m, 2H, H*_h/j_*), 2.43 (br. m, 4H, 4 of H*_I_*), 2.07 (br. m, 4H, 4 of H*_I_*), 1.64–1.56 (m, 4H, H*_I’_*), 1.34 (s, 27H, H*_o_*), 1.34–1.10 (m, 12H, H*_J’_*, H*_K’_*, H*_L’_*, H*_i_*), 1.17 (s, 36H, H*_a_*). ^13^C-NMR (CDCl_3_, 126 MHz, 298 K) δ: 160.1, 159.3, 158.7, 158.3, 156.1, 155.6, 155.3, 151.3, 148.4, 145.8, 144.5, 139.5, 137.5, 137.4, 137.3, 132.8, 131.9, 130.9, 130.6, 129.9, 129.8, 129.2, 127.8, 124.3, 122.3, 122.1, 121.9, 121.7, 121.1, 120.5, 120.0 (×2), 119.2, 115.1, 114.7, 114.4, 112.7, 73.2, 72.5, 72.4, 70.3, 67.6, 66.4, 63.9, 63.2, 47.0, 35.1, 34.5, 31.6, 31.5, 30.3, 29.6, 29.1, 29.0, 25.9, 25.0. ESI-MS *m/z* = 1366.27 [M + 2H]^2+^ calc. 1366.28.

## 4. Conclusions

We have shown that the steric encumbrance of a macrocycle can be used in lieu of protecting group chemistry to successively prepare [2]-, [3]- and [4]rotaxanes from a simple triethynylbenzene unit. Whilst the first two iterations proceed in good yield, the steric bulk of two macrocycles results in severely diminished yields of the third AT-CuAAC reaction. However, through alleviation of steric constraints by employing larger and more flexible macrocycles and stoppering units the yield of this final iteration could be raised from <5% to 27%. We are currently investigating structures that will allow the high yielding synthesis of [4]rotaxanes, whilst maintaining the selectivity for a single AT-CuAAC reaction with each step.

## Supplementary Materials

Supplementary materials can be accessed at: http://www.mdpi.com/1420-3049/22/1/89/s1. ^1^H-, ^13^C- and 2D-NMR and MS spectral data and crystallographic data. X-ray crystallographic data for **8** are available in CIF format from the CCDC (1517622).
