# Supplementary material for: Stepwise, Protecting Group Free Synthesis of [4]Rotaxanes
Source: Molecules. 2017 Jan 9;22(1):89. doi: 10.3390/molecules22010089 (PMC6155830; doi:10.3390/molecules22010089)
Supplement: Supplementary file 1 [file molecules-22-00089-s001.pdf]

Supplementary Materials: Stepwise, Protecting Group Free Synthesis of [4]Rotaxanes

James E. M. Lewis, Joby Winn and Stephen M. Goldup

General Experimental

Synthesis: Unless otherwise stated, all reagents, including anhydrous solvents, were purchased from commercial sources and used without further purification. All reactions were carried out under an atmosphere of N_2 using anhydrous solvents unless otherwise stated. Petrol refers to the fraction of petroleum ether boiling in the range 40–60 °C. EDTA-NH_3 solution refers to an aqueous solution of NH_3 (17% *w*/*w*) saturated with sodium-ethylenediaminetetraacetate. Flash column chromatography was performed using Biotage Isolera-4 automated chromatography system, employing Biotage SNAP or ZIP cartridges. Analytical TLC was performed on precoated silica gel plates (0.25 mm thick, 60F254, Merck, Darmstadt, Germany) and observed under UV light. Microwave reactions were completed using a CEM Discover S Microwave system. Reactions were run at a power level of 150 W.

Analysis: NMR spectra were recorded on Bruker AV400, AV3-400, AV500 or Bruker AV600 instrument, at a constant temperature of 298 K. Chemical shifts are reported in parts per million from low to high field and referenced to residual solvent. Coupling constants (*J*) are reported in Hertz (Hz). Standard abbreviations indicating multiplicity were used as follows: m = multiplet, quint = quintet, q = quartet, t = triplet, d = doublet, s = singlet, app. = apparent, br = broad. Signal assignment was carried out using 2D NMR methods (HSQC, HMBC, COSY, NOESY) where necessary. In the case of some complex multiplets with contributions from more than one signal absolute assignment was not possible. Here indicative either/or assignments (e.g., $H_{A/B}$ for H_A or H_B) are provided. All melting points were determined using a Griffin apparatus. Low resolution mass spectrometry was carried out by the mass spectrometry services at the University of Southampton (Waters TQD mass spectrometer equipped with a triple quadrupole analyser with UHPLC injection [BEH C18 column; MeCN-hexane gradient {0.2% formic acid}]). High resolution mass spectrometry was carried out by the mass spectrometry services at the University of Southampton (MaXis, Bruker Daltonics, with a Time of Flight (TOF) analyser; samples were introduced to the mass spectrometer via a Dionex Ultimate 3000 autosampler and uHPLC pump in a gradient of 20% acetonitrile in hexane to 100% acetonitrile (0.2% formic acid) over 5 min at 0.6 mL min; column: Acquity UPLC BEH C18 (Waters) 1.7 micron 50 × 2.1mm).

The Following Compounds Were Synthesized According to Literature Procedures:

1-Azido-3,5-di-*tert*-butylbenzene (**2**), 1,3,5-triethynylbenzene (**6**), azide stopper **10** and macrocycles **3** and **9**.

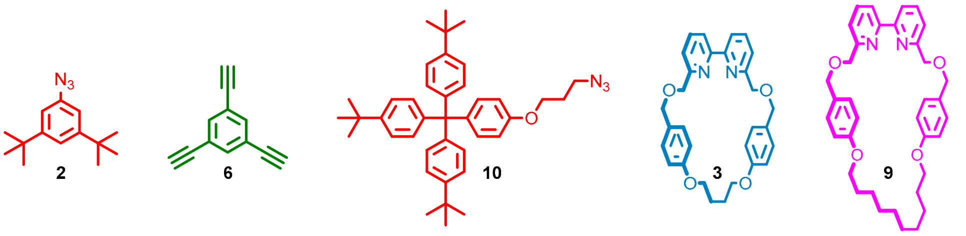

General Procedure

$^iPr_2NEt$ (0.1 M in EtOH, 1 eq.) was added to a solution of **macrocycle** (1 eq.), $[Cu(CH_3CN)_4]PF_6$ (1 eq.), **alkyne** (1 eq.) and **azide** (1 eq.) in EtOH (2.5 mL) in a CEM vial. The deep red reaction mixture was stirred at 100 °C under microwave irradiation for 2 h. After removal of the solvent in vacuo the residue was dissolved in CH_2Cl_2 (2.5 mL) and stirred at 100 °C under microwave irradiation for 1 h. To the cooled reaction mixture was added EDTA-NH_3 solution (50 mL) and extracted with CH_2Cl_2 (3

× 50 mL). The combined organic extracts were dried ($MgSO_4$) and the solvent removed *in vacuo*. Purification by flash column chromatography on silica gel yielded the product.

Experimental Procedures

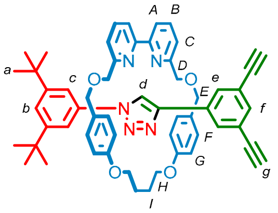

[2]Rotaxane 7

General procedure. **6** (22.5 mg, 0.15 mmol, 1 eq.), **2** (34.7 mg, 0.15 mmol, 1 eq.), **3** (72.3 mg, 0.15 mmol, 1 eq.), $[Cu(CH_3CN)_4](PF_6)$ (55.9 mg, 0.15 mmol, 1 eq.). Purification by column chromatography on silica gel (petrol with 0% to 100% gradient of Et_2O) yielded the product as a white solid (101 mg, 78%). m.p. = 171–174 °C. ^{1}H-NMR (600 MHz, $CDCl_3$) δ: 10.22 (s, 1H, H_d), 7.74–7.71 (m, 4H, H_B, H_e), 7.63 (d, J = 7.8, 2H, H_A), 7.52 (d, J = 1.6, 2H, H_c), 7.41 (d, J = 7.7, 2H, H_C), 7.33 (t, J = 1.4, 1H, H_f), 7.27 (t, J = 1.6, 1H, H_b), 6.65 (d, J = 8.5, 4H, H_F), 6.53 (d, J = 8.5, 4H, H_G), 4.52–4.48 (m, 4H, H_H), 4.37–4.32 (m, 4H, H_E), 3.91–3.86 (m, 4H, H_D), 2.93 (s, 2H, H_g), 2.29–2.25 (m, 4H, H_I), 1.21 (s, 18H, H_a). ^{13}C NMR (151 MHz, $CDCl_3$) δ: 159.4, 159.2, 155.9, 151.8, 144.0, 137.4, 137.1, 133.6, 132.1, 130.0, 129.1, 128.0, 121.9, 121.0, 120.3, 115.2, 114.6, 83.1, 77.2 (determined by HSQC), 73.1, 70.4, 66.5, 35.2, 31.4, 24.9. ESI-MS m/z = 864.4484 $[M + H]^+$ calc. 864.4483.

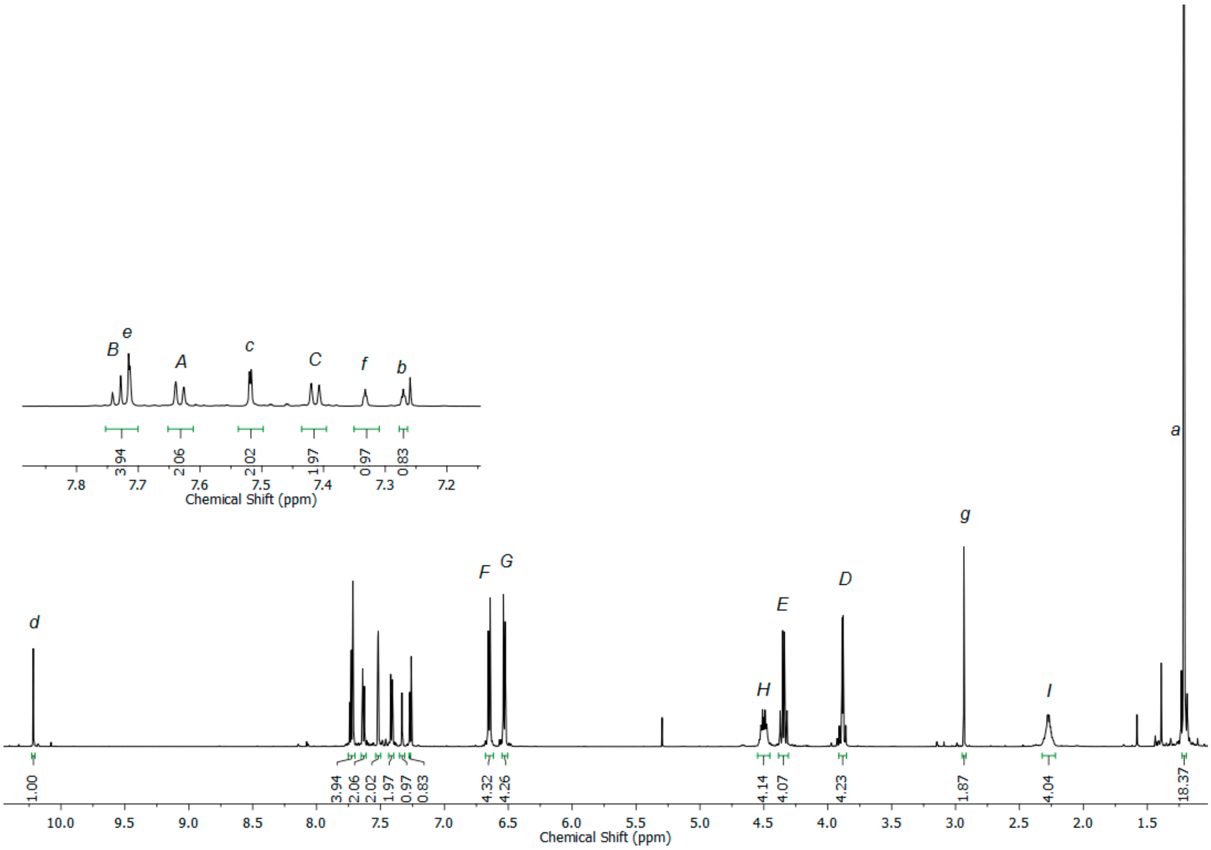

Figure S1. ^{1}H-NMR ($CDCl_3$, 600 MHz) of **7**.

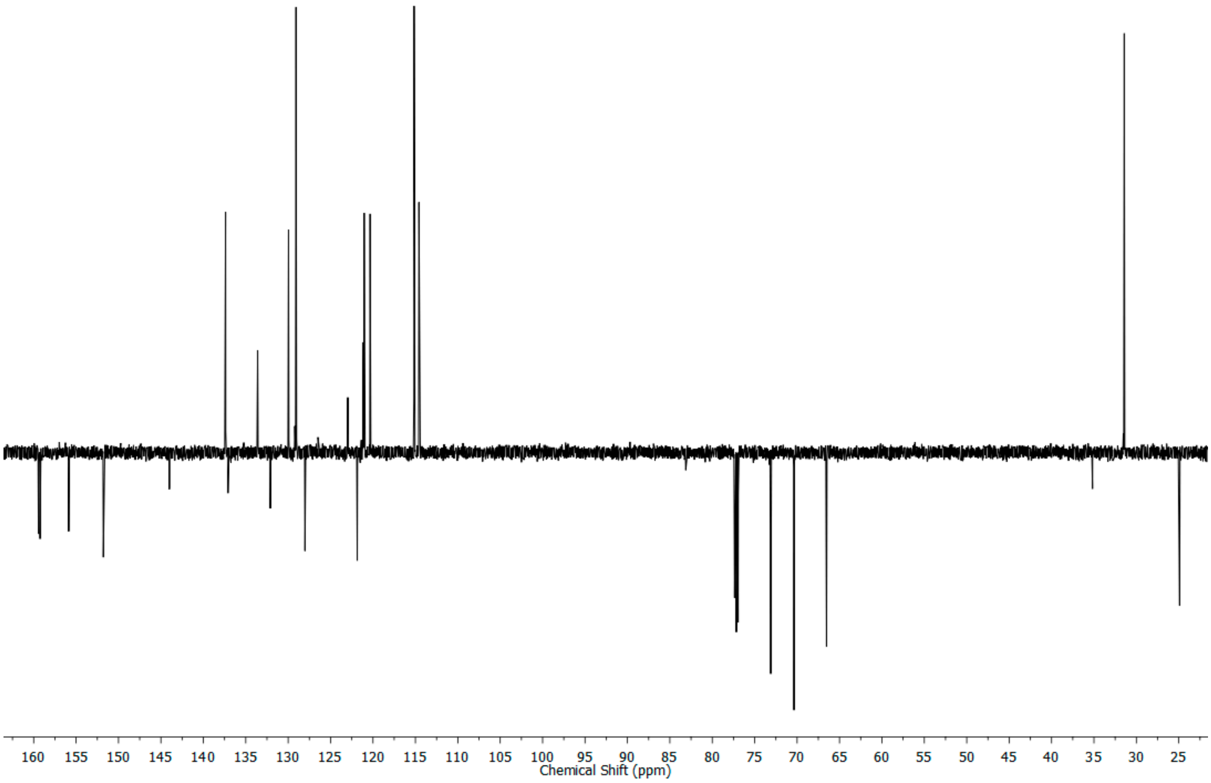

Figure S2. DEPTQ NMR ($CDCl_3$, 151 MHz) of **7**.

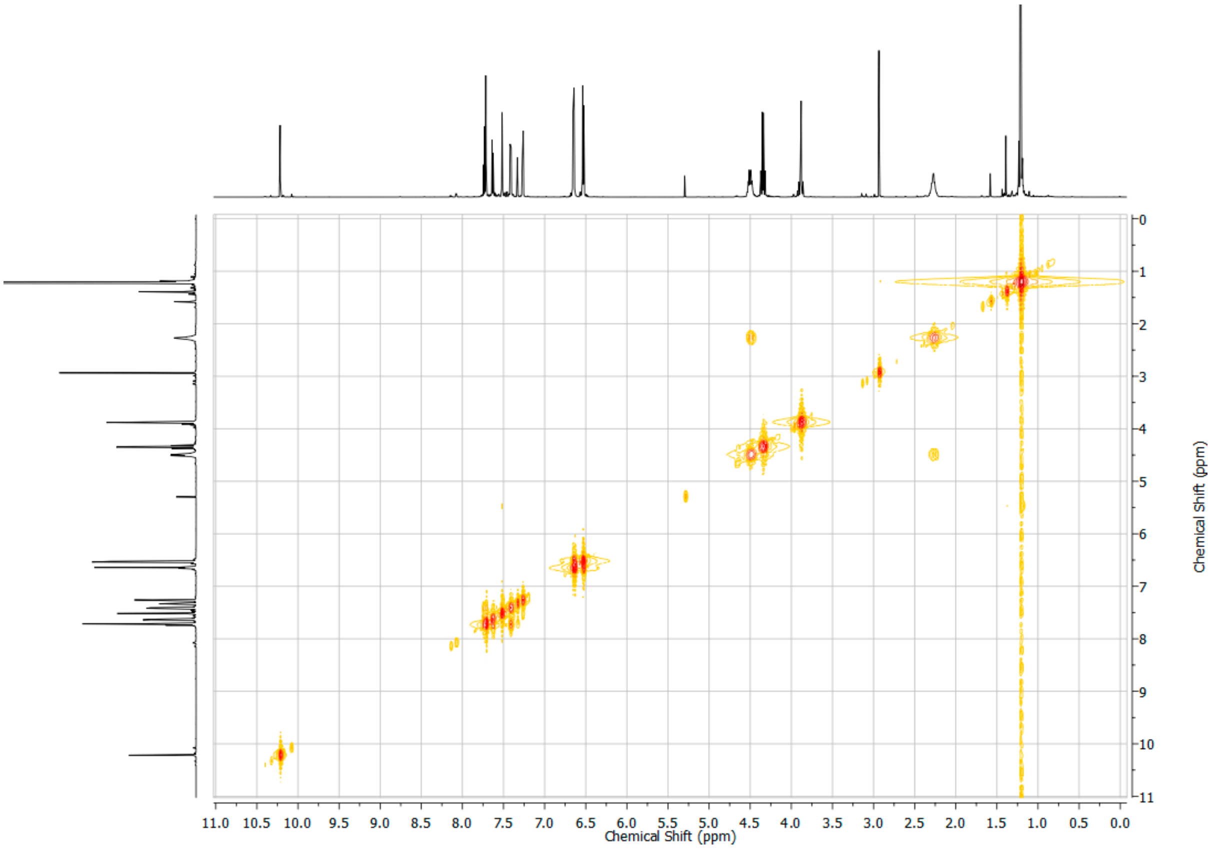

Figure S3. COSY NMR ($CDCl_3$) of **7**.

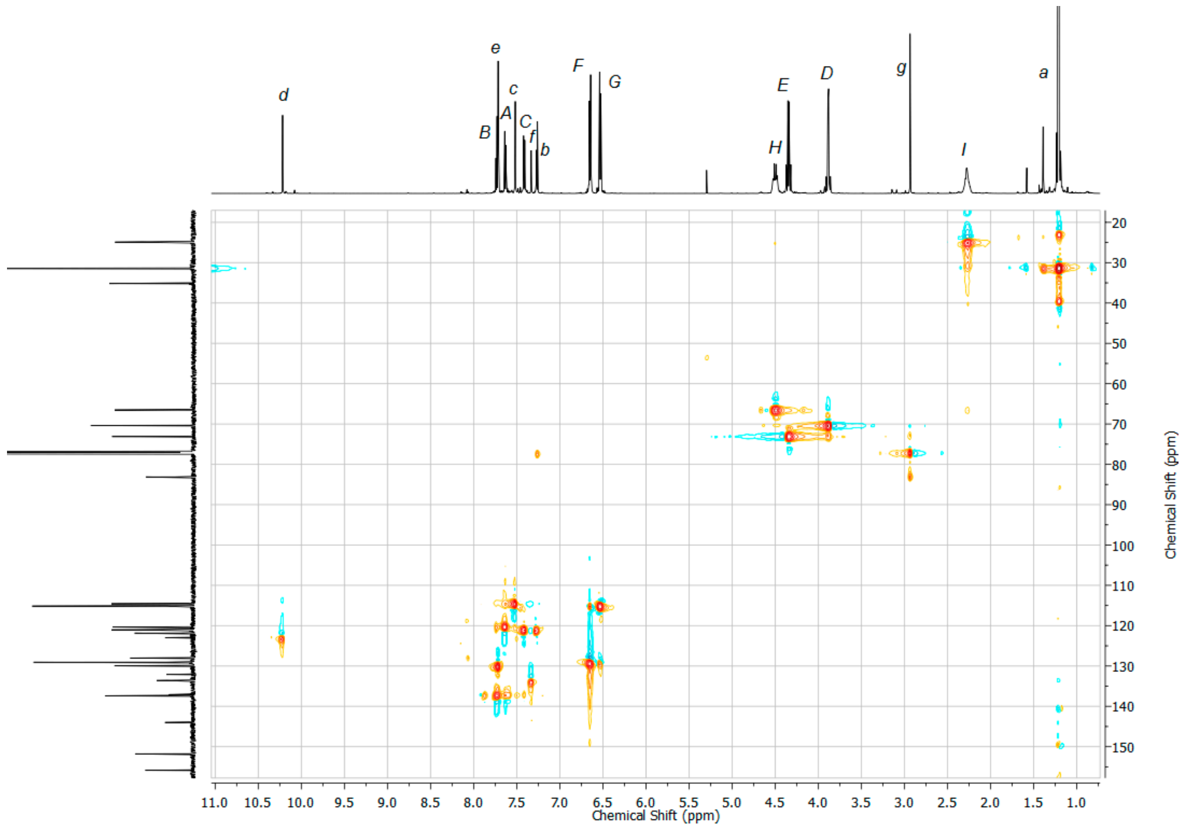

Figure S4. HSQC NMR ($CDCl_3$) of **7**.

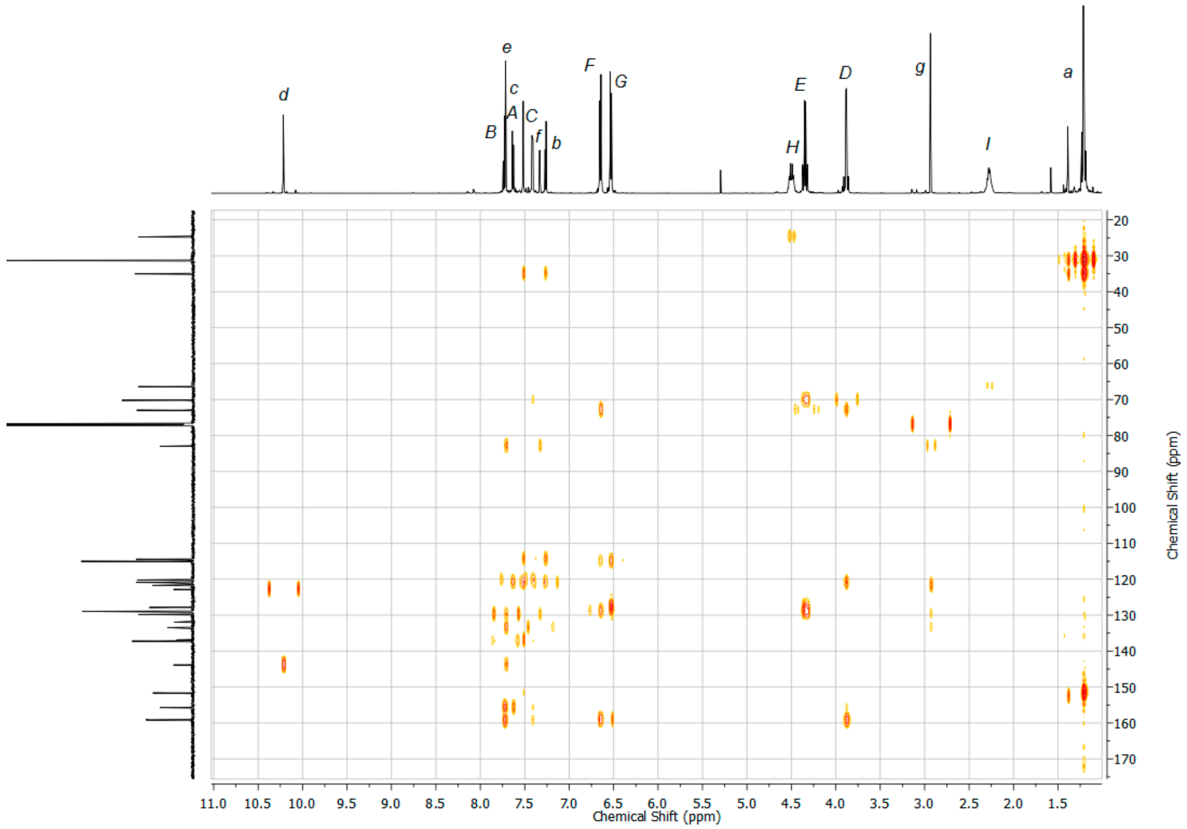

Figure S5. HMBC NMR ($CDCl_3$) of **7**.

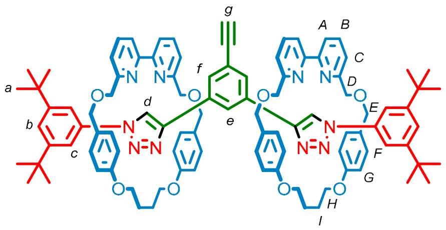

[3]Rotaxane 8

General procedure. **7** (60.4 mg, 0.069 mmol, 1 eq.), **2** (16.0 mg, 0.069 mmol, 1 eq.), **3** (33.3 mg, 0.069 mmol, 1 eq.), $[Cu(CH_3CN)_4](PF_6)$ (25.3 mg, 0.069 mmol, 1 eq.). Purification by column chromatography on silica gel (petrol with 0 to 100% gradient of Et_2O) yielded the product as a white solid (65 mg, 60%). m.p. = 226–228 °C. ^{1}H-NMR (400 MHz, $CDCl_3$) δ: 10.33 (s, 2H, H_d), 8.76 (s, 1H, H_e), 7.74 (t, *J* = 7.7, 4H, H_B), 7.68 (d, *J* = 7.4, 4H, H_A), 7.45 (d, *J* = 7.6, 4H, H_C), 7.43 (d, *J* = 1.6, 4H, H_c), 7.40 (d, *J* = 1.5, 2H, H_f), 7.21 (t, *J* = 1.5, 2H, H_b), 6.63 (d, *J* = 8.5, 8H, H_F), 6.49 (d, *J* = 8.5, 8H, H_G), 4.66 (app. q, *J* = 7.4, 4H, H_H), 4.46 (d, *J* = 12.2, 4H, 4 of H_E), 4.27 (d, *J* = 12.2, 4H, 4 of H_E), 4.21–4.15 (m, 4H, 4 of H_H), 3.97 (s, 8H, H_D), 2.61 (s, 1H, H_g), 2.40–2.33 (m, 4H, 4 of H_I), 2.17–2.09 (m, 4H, 4 of H_I), 1.19 (s, 36H, H_a). ^{13}C NMR (101 MHz, $CDCl_3$) δ: 159.7, 159.3, 155.6, 151.4, 145.1, 137.4, 137.2, 132.1, 129.0, 128.5, 127.9, 121.0, 120.7, 120.2, 115.2, 114.8, 84.2, 75.6, 73.1, 70.4, 66.5, 35.1, 31.5, 25.1. ESI-MS *m*/*z* = 1577.87 $[M + H]^+$ calc. 1577.84; 789.44 $[M + 2H]^{2+}$ calc. 789.42.

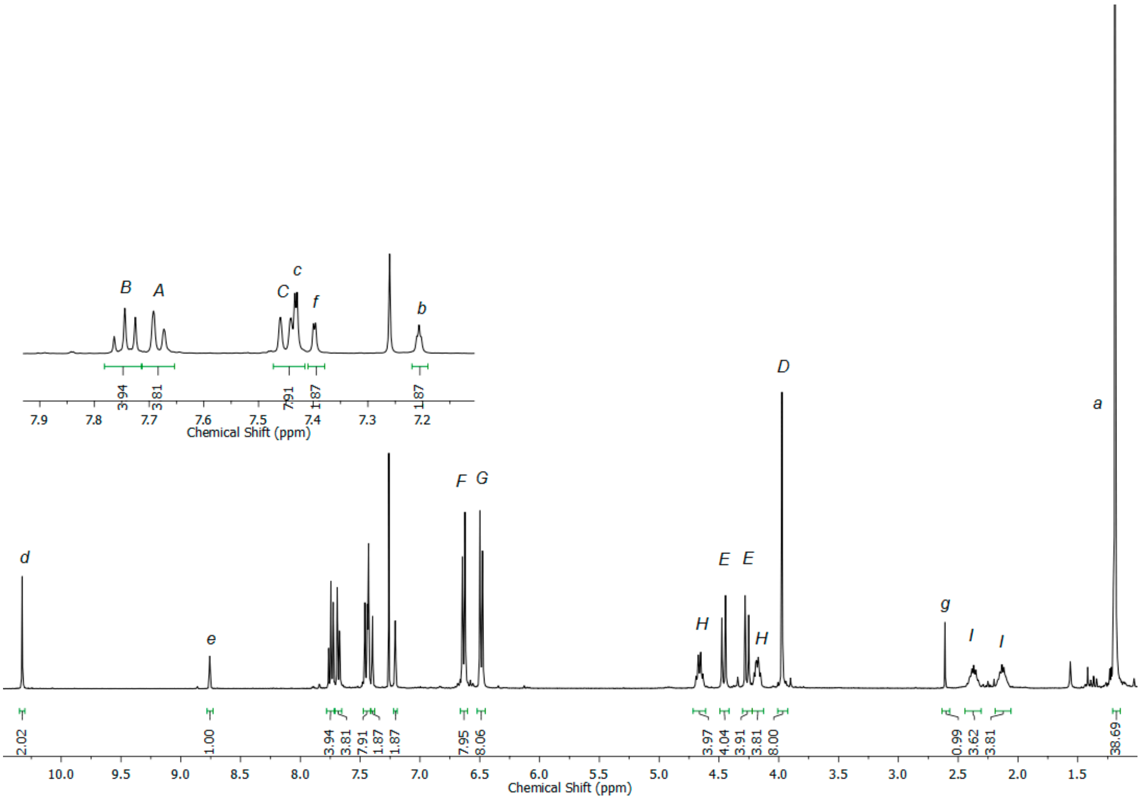

Figure S6. ^{1}H-NMR ($CDCl_3$, 400 MHz) of **8**.

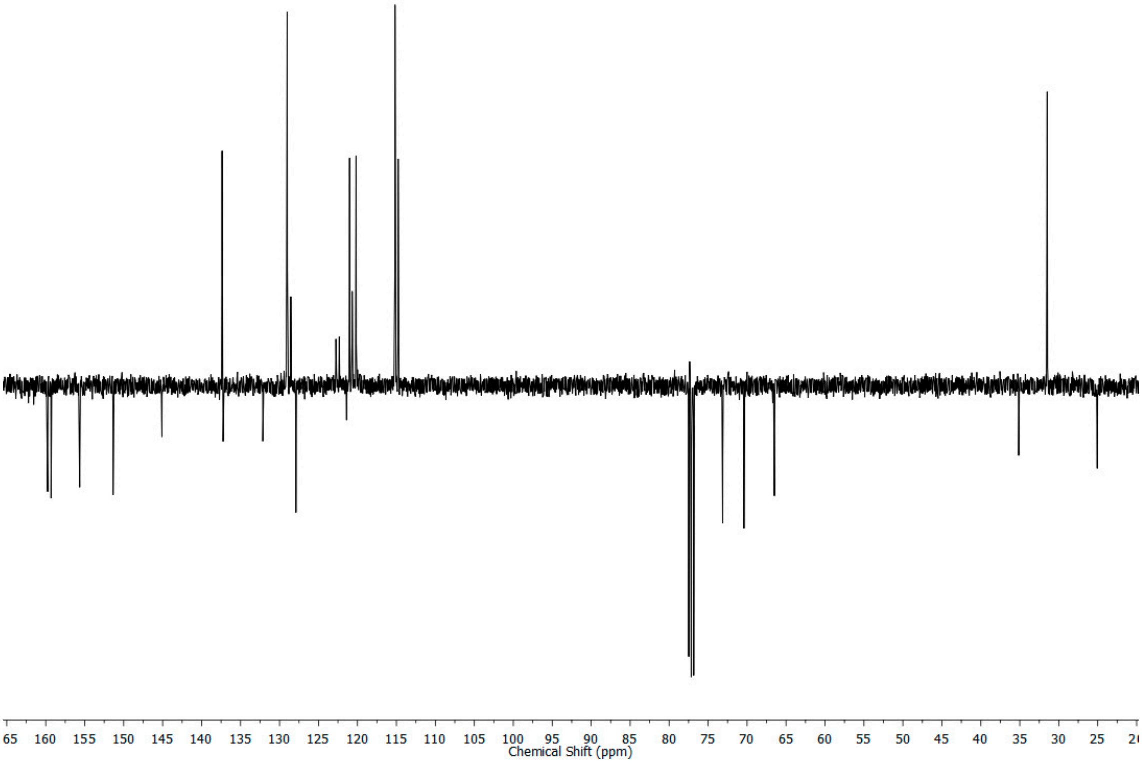

Figure S7. DEPTQ NMR ($CDCl_3$, 151 MHz) of **8**.

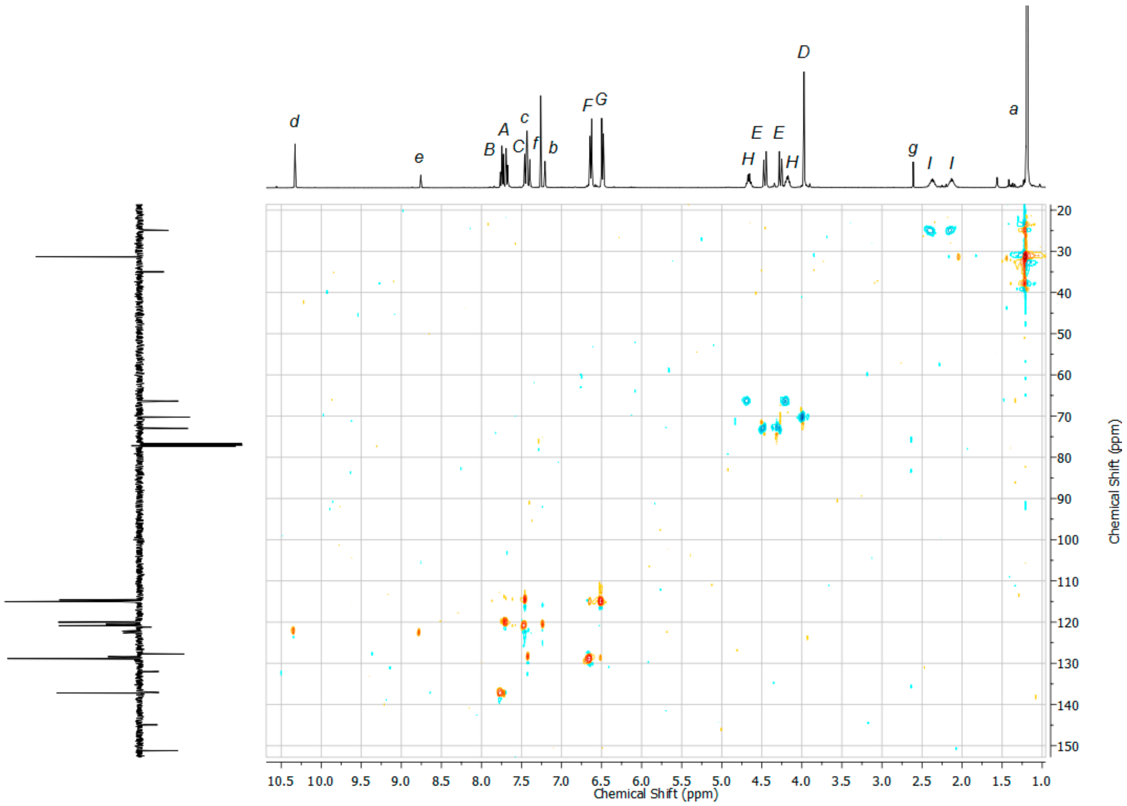

Figure S8. HSQC NMR ($CDCl_3$) of **8**.

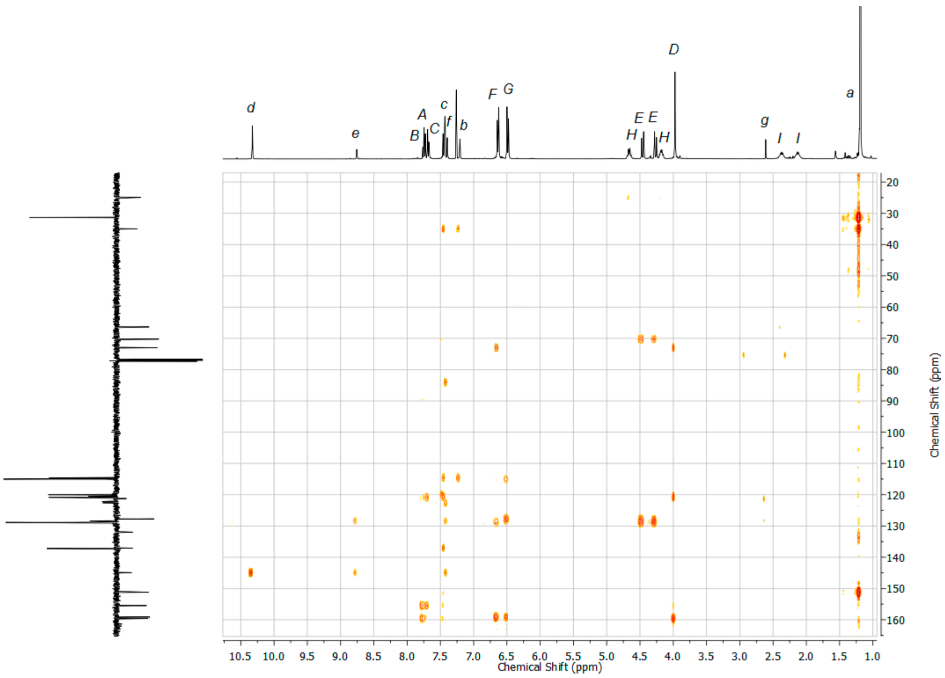

Figure S9. HMBC NMR ($CDCl_3$) of **8**.

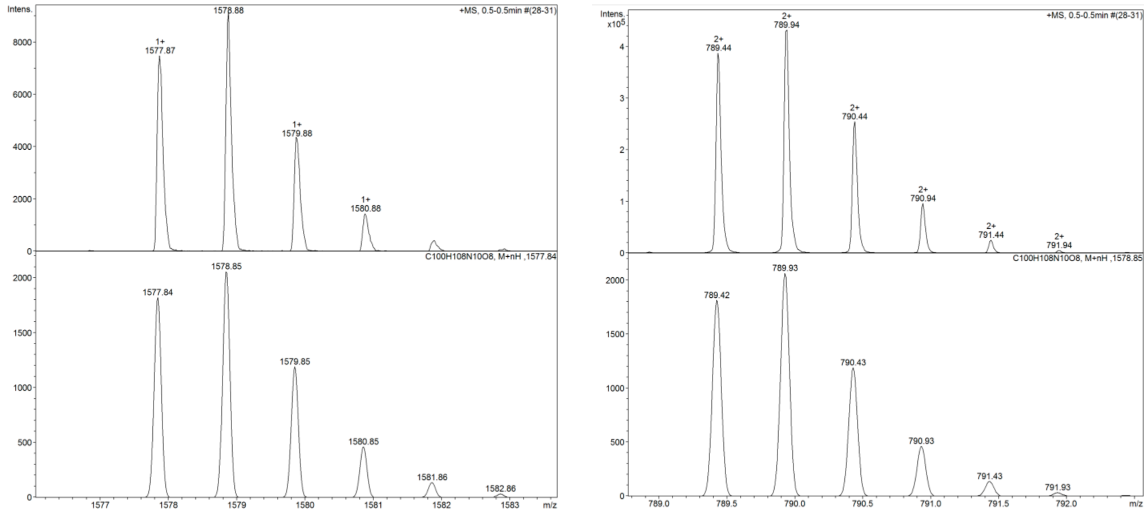

Figure S10. ESI-MS isotopic patterns of 8; observed (**left**) and calculated (**right**).

General procedure. **8** (16.1 mg), **2** (4.9 mg, 0.010 mmol, 1 eq.), **3** (4.9 mg, 0.010 mmol, 1 eq.), $[Cu(CH_3CN)_4](PF_6)$ (3.7 mg, 0.010 mmol, 1 eq.). Purification by column chromatography on silica gel (petrol with 0 to 100% gradient of Et_2O) yielded the **11a** as a white foam (10.2 mg, 55%).

[3]Rotaxane 11a

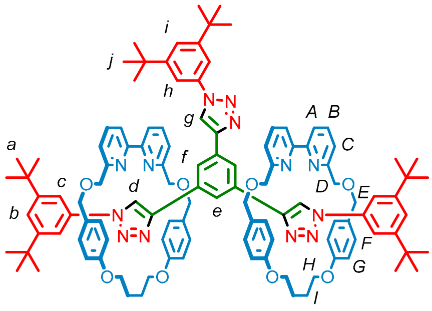

^1H-NMR ($CDCl_3$, 400 MHz, 298 K) δ: 10.33 (s, 2H, H_d), 8.72 (br. m, 1H, H_e), 8.06 (d, *J* = 1.5, 2H, H_f), 7.59 (app. t, *J* = 7.7, 4H, H_B), 7.54–7.51 (m, 5H, H_A, H_i), 7.48 (s, 1H, H_g), 7.41 (d, *J* = 1.6, 4H, H_c), 7.39 (d, *J* = 7.5, 4H, H_C), 7.32 (d, *J* = 1.7, 2H, H_h), 7.20 (t, *J* = 1.5, 2H, H_b), 6.70 (d, *J* = 8.5, 8H, H_F), 6.54 (d, *J* = 8.5, 8H, H_G), 4.79–4.73 (m, 4H, 4 of H_H), 4.50 (d, *J* = 12.2, 4H, 4 of H_E), 4.28 (d, *J* = 12.1, 4H, 4 of H_E), 4.20–4.15 (m, 4H, 4 of H_H), 4.07–4.00 (m, 8H, H_D), 2.43 (br. m, 4H, 4 of H_I), 2.12 (br. m, 4H, 4 of H_I), 1.41 (s, 18H, H_j), 1.18 (s, 36H, H_a). ^{13}C-NMR ($CDCl_3$, 101 MHz, 298 K) δ: 159.8, 159.4, 155.5, 152.6, 151.3, 148.7, 145.8, 137.4, 137.2, 137.2, 132.6, 130.3, 129.2, 127.9, 122.9, 122.7, 122.3, 122.2, 121.1, 120.6, 120.2, 118.5 (via HSQC), 115.9, 115.2, 114.8, 73.2, 70.4, 66.6, 35.3, 35.1, 31.6, 31.5, 25.1. ESI-MS *m*/*z* = 905.01 $[M + 2H]^{2+}$ calc. 905.01.

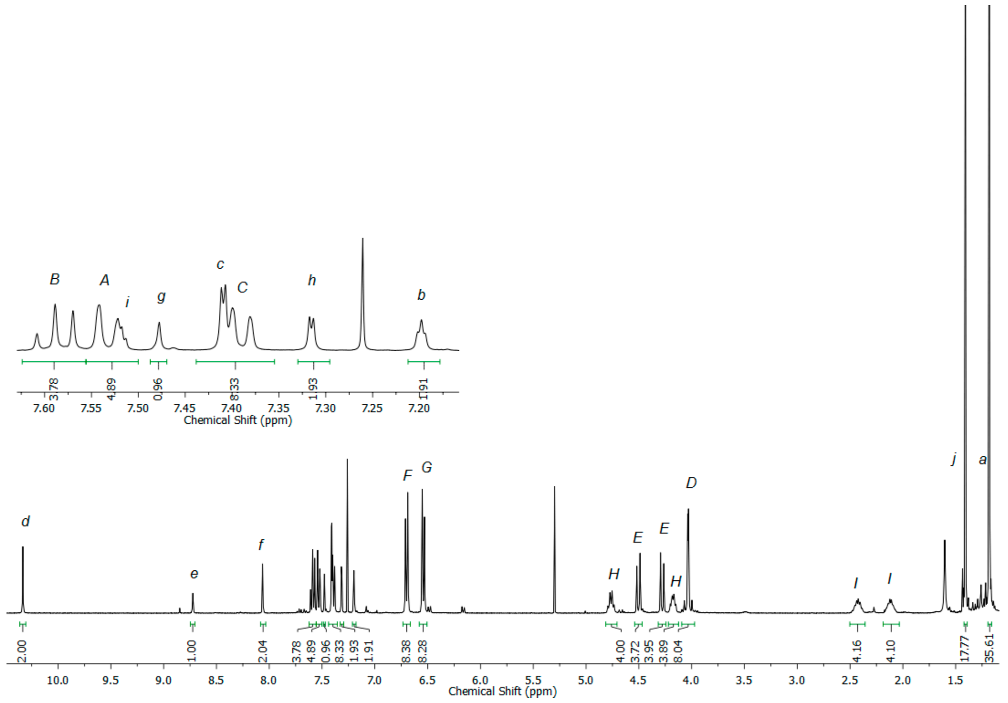

Figure S11. ^1H-NMR ($CDCl_3$, 400 MHz) of **11a**.

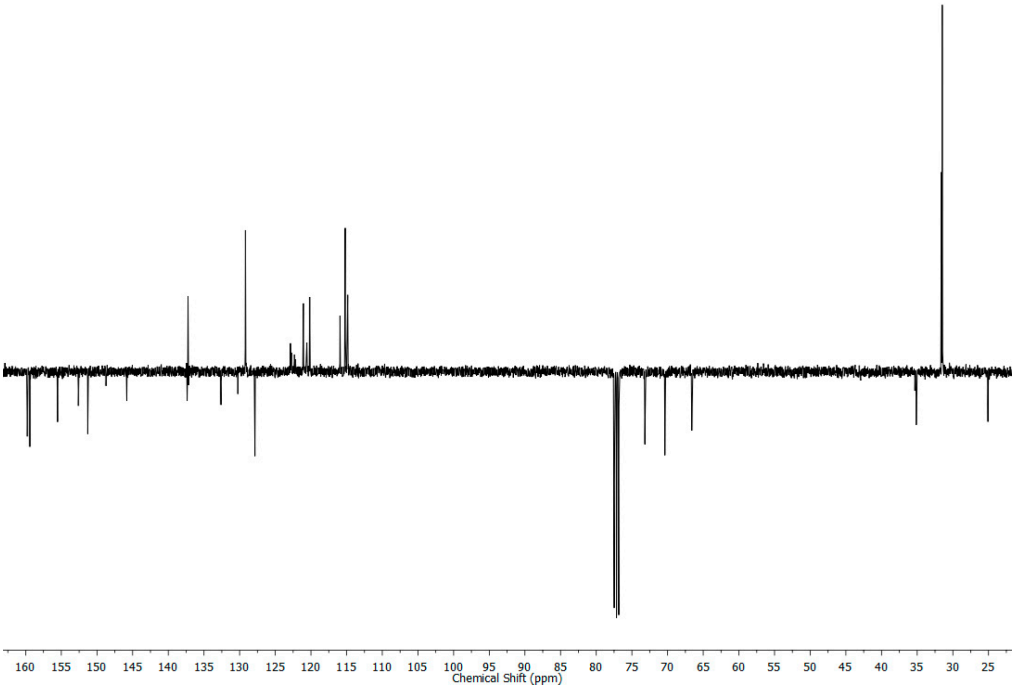

Figure S12. JMOD NMR ($CDCl_3$, 101 MHz) of **11a**.

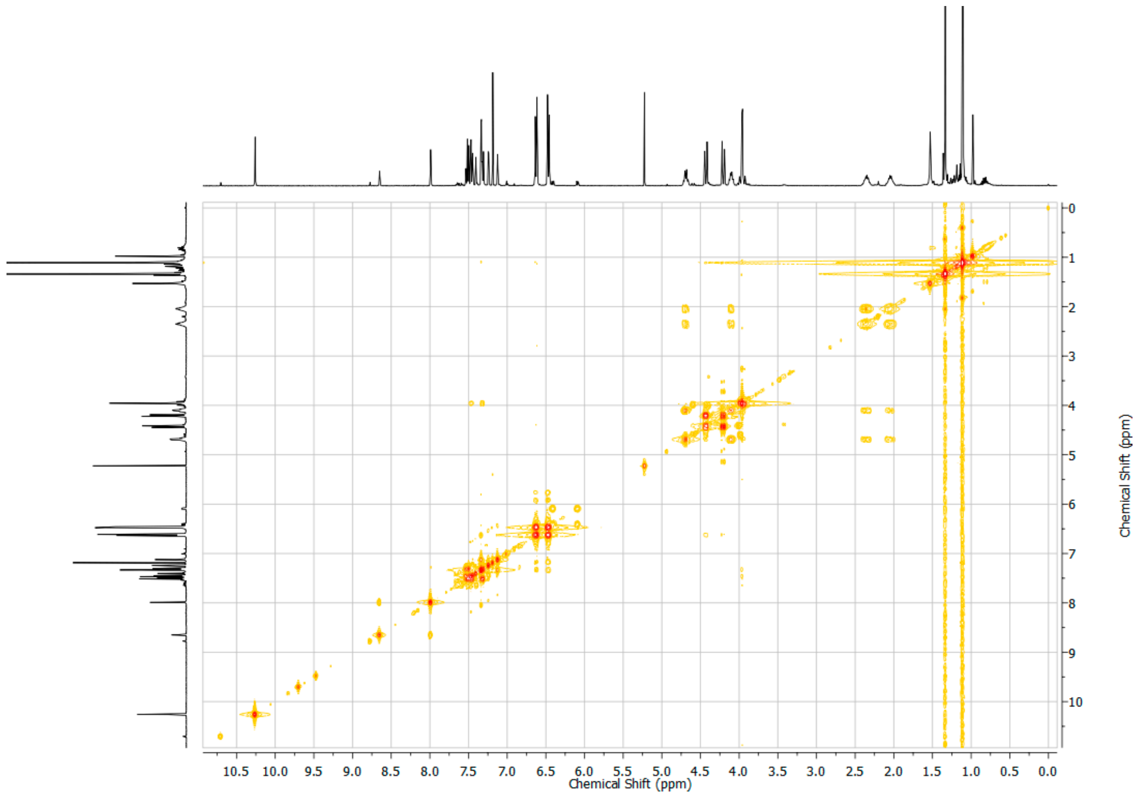

Figure S13. COSY NMR ($CDCl_3$) of **11a**.

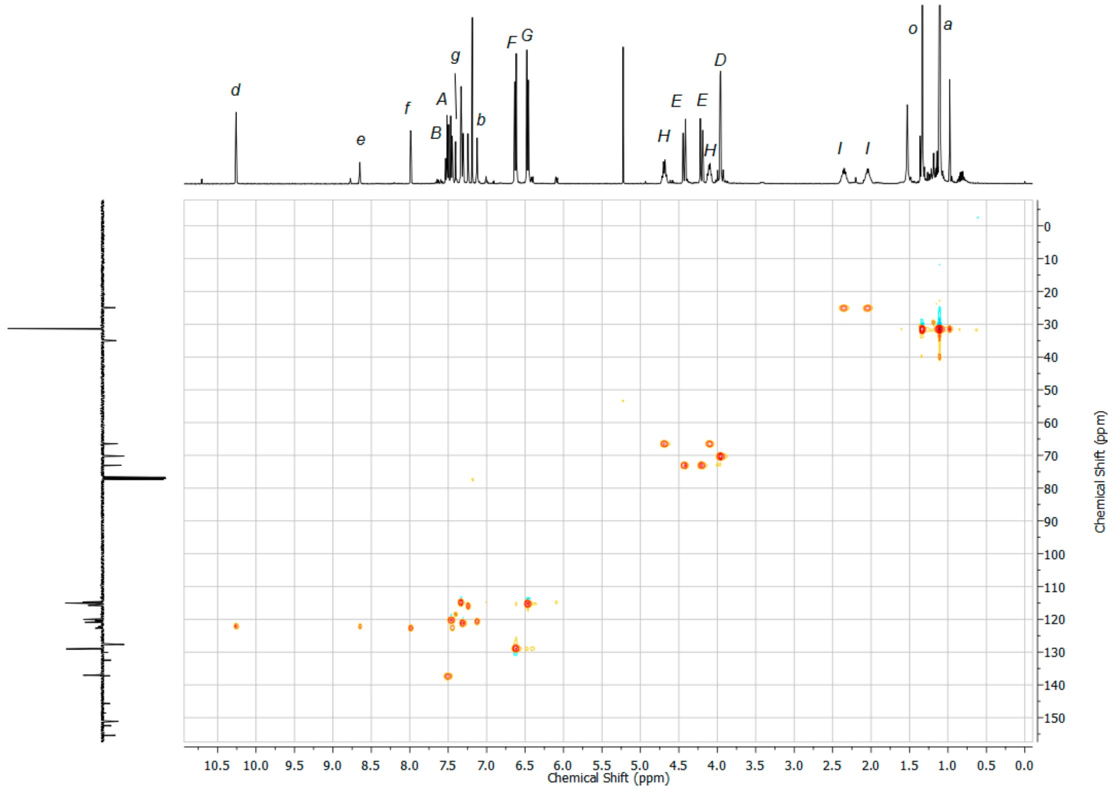

Figure S14. HSQC NMR ($CDCl_3$) of **11a**.

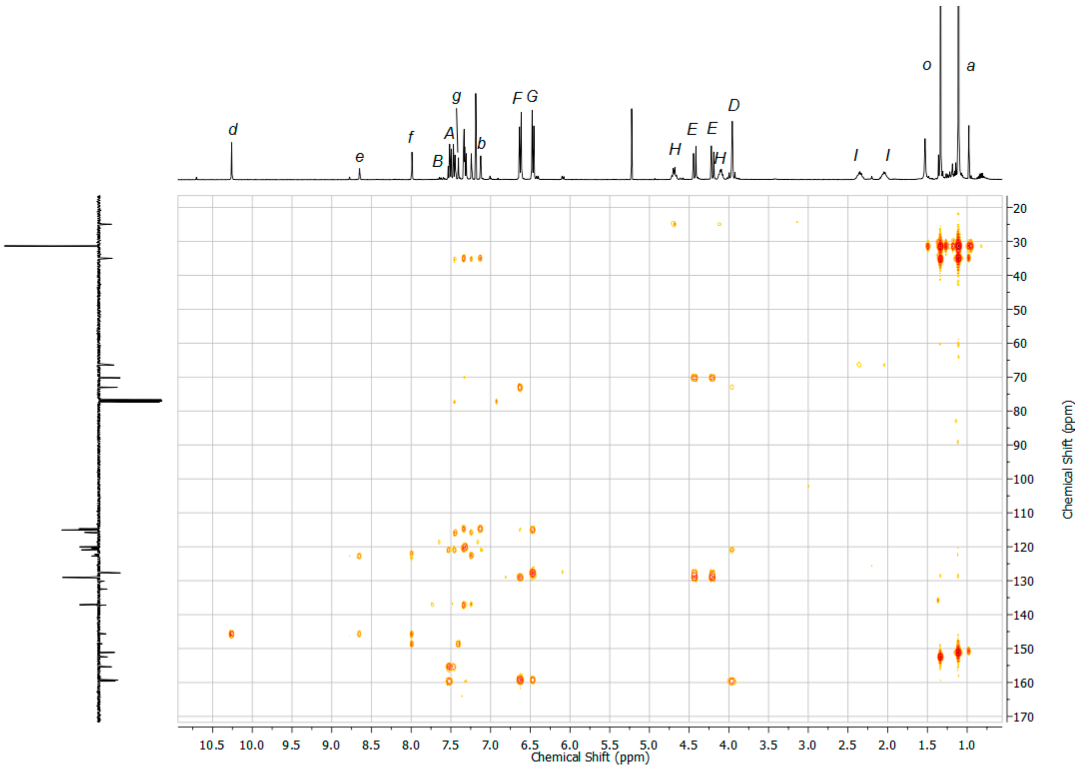

Figure S15. HMBC NMR ($CDCl_3$) of **11a**.

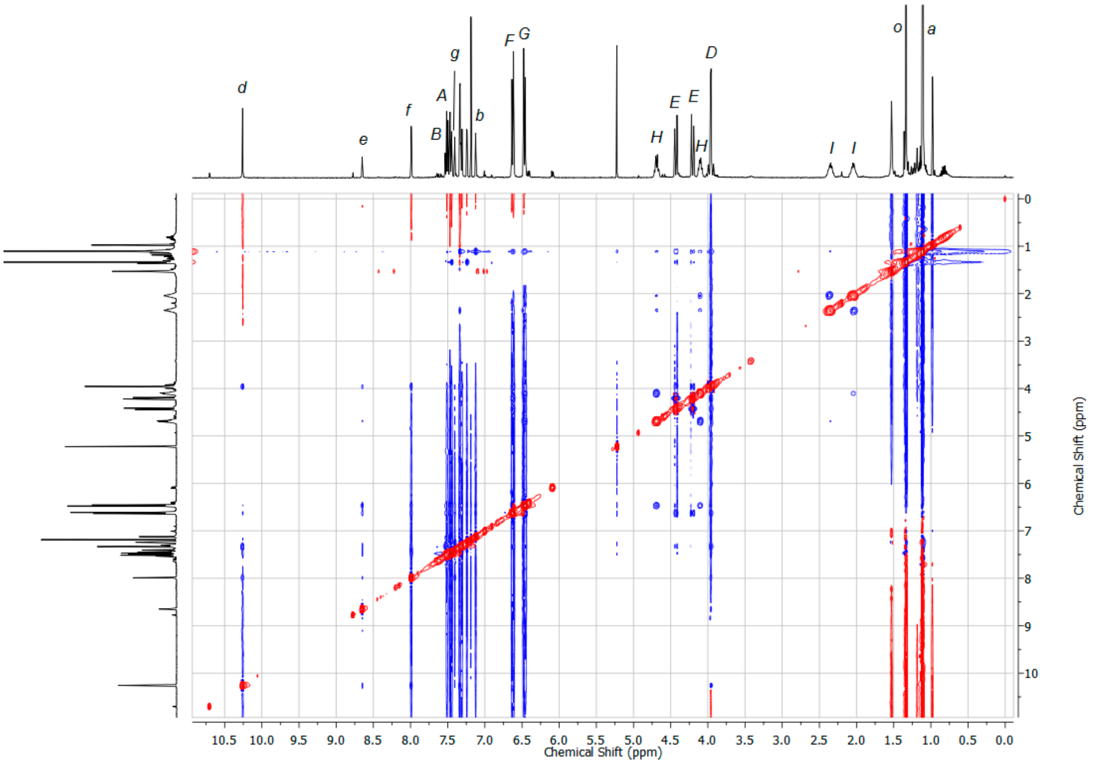

Figure S16. NOESY NMR ($CDCl_3$) of **11a**.

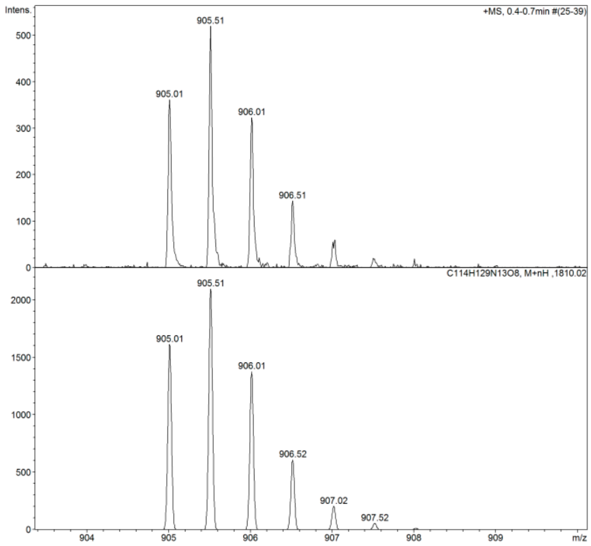

Figure S17. ESI-MS isotopic pattern of 11a; observed (**top**) and calculated (**bottom**).

General procedure. **8** (17.5 mg, 0.011 mmol, 1 eq.), **10** (6.5 mg, 0.011 mmol, 1 eq.), **3** (5.4 mg, 0.011 mmol, 1 eq.), $[Cu(CH_3CN)_4](PF_6)$ (4.0 mg, 0.011 mmol, 1 eq.). Purification by column chromatography on silica gel (petrol with 0 to 100% gradient of Et_2O) yielded **11b** (17.5 mg, 73%) and **12b** (4.7 mg, 16%) as white foams.

[3]Rotaxane 11b

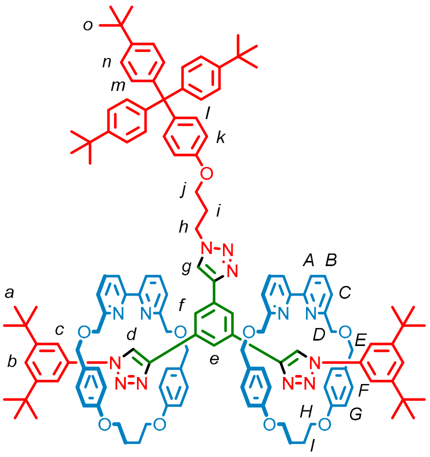

^{1}H-NMR ($CDCl_3$, 400 MHz, 298 K) δ: 10.44 (s, 2H, H$_d$), 8.80 (s, 1H, H$_e$), 8.15 (d, *J* = 0.8, 2H, H$_f$), 7.71–7.68 (m, 8H, H$_A$, H$_B$), 7.44–7.42 (m, 4H, H$_C$), 7.38 (d, *J* = 1.3, 4H, H$_c$), 7.24 (d, *J* = 8.5, 6H, H$_m$ or H$_n$), 7.19–7.17 (m, 3H, H$_b$, H$_l$), 7.11 (d, *J* = 8.5, 6H, H$_m$ or H$_n$), 6.80 (d, *J* = 8.8, 2H, H$_k$), 6.68–6.65 (m, 9H, H$_F$, H$_g$), 6.52 (d, *J* = 8.5, 8H, H$_G$), 4.79 (app. q, *J* = 7.6, 4H, 4 of H$_H$), 4.53 (d, *J* = 12.2, 4H, 4 of H$_E$), 4.22–4.12 (m, 10H, 4 of H$_E$, 4 of H$_H$, H$_h$), 4.05–3.98 (m, 8H, H$_D$), 3.87 (t, *J* = 5.5, 2H, H$_j$), 2.45 (br. m, 4H, 4 of H$_I$), 2.10 (br. m, 6H, 4 of H$_I$, H$_i$), 1.30 (s, 27H, H$_o$), 1.17 (s, 36H, H$_a$). ^{13}C-NMR ($CDCl_3$, 101 MHz, 298 K) δ: 160.1, 159.4, 156.5, 155.3, 151.3, 148.6, 148.5, 145.8, 144.2, 140.5, 137.4, 137.4, 132.7, 132.6, 130.9, 130.4, 129.2, 127.8, 125.7, 124.3, 122.4, 122.1, 122.0, 121.2, 120.5, 120.0, 115.1, 114.7, 113.1, 73.2, 70.3, 66.5, 64.3, 63.2, 47.0, 35.1, 34.5, 31.5, 31.5, 30.3, 25.1. ESI-MS *m*/*z* = 1083.12 $[M + 2H]^{2+}$ calc. 1083.12.

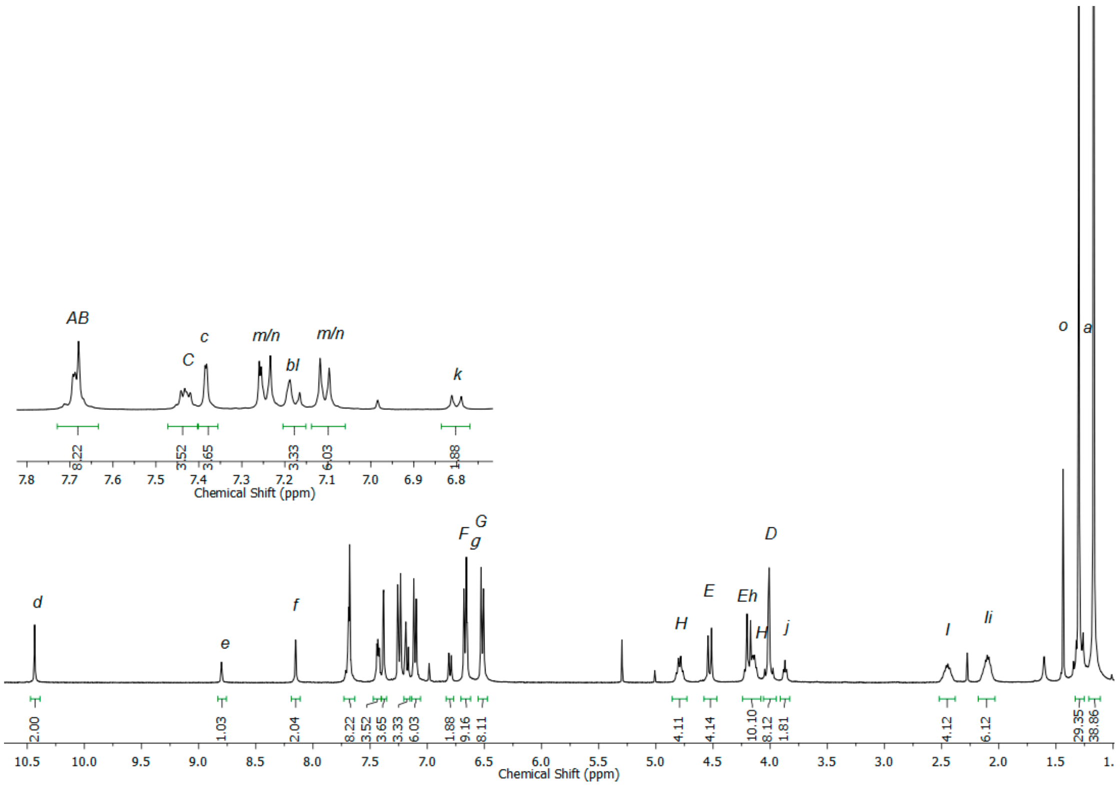

Figure S18. ^{1}H-NMR ($CDCl_3$, 400 MHz) of **11b**.

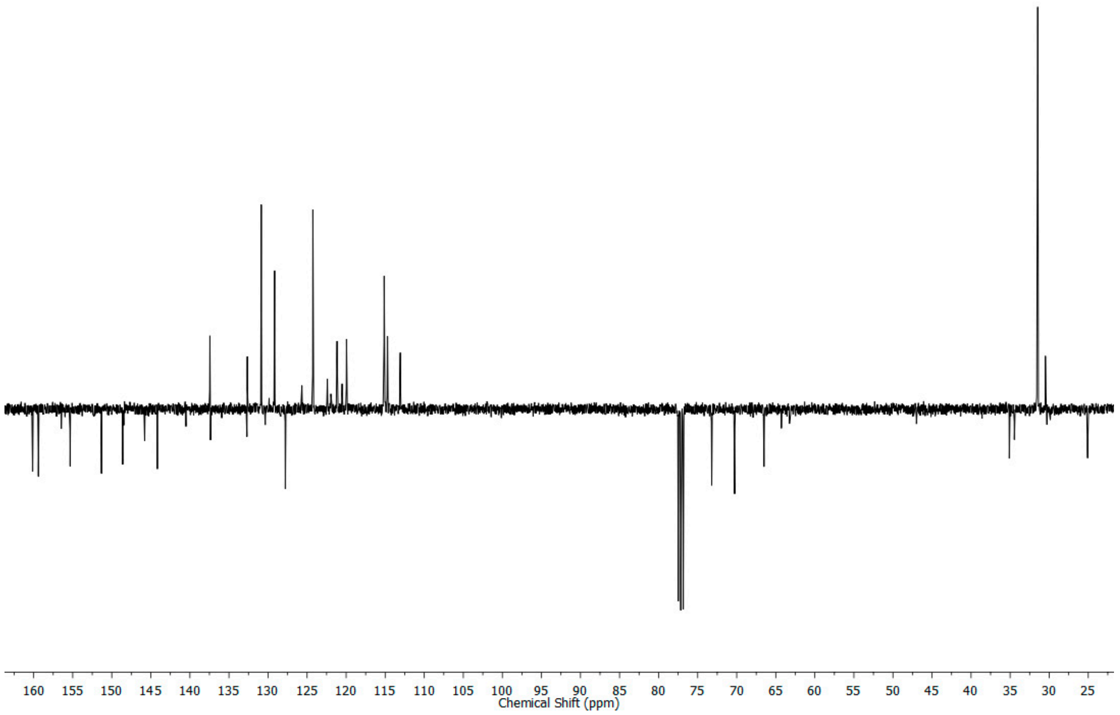

Figure S19. JMOD NMR ($CDCl_3$, 101 MHz) of **11b**.

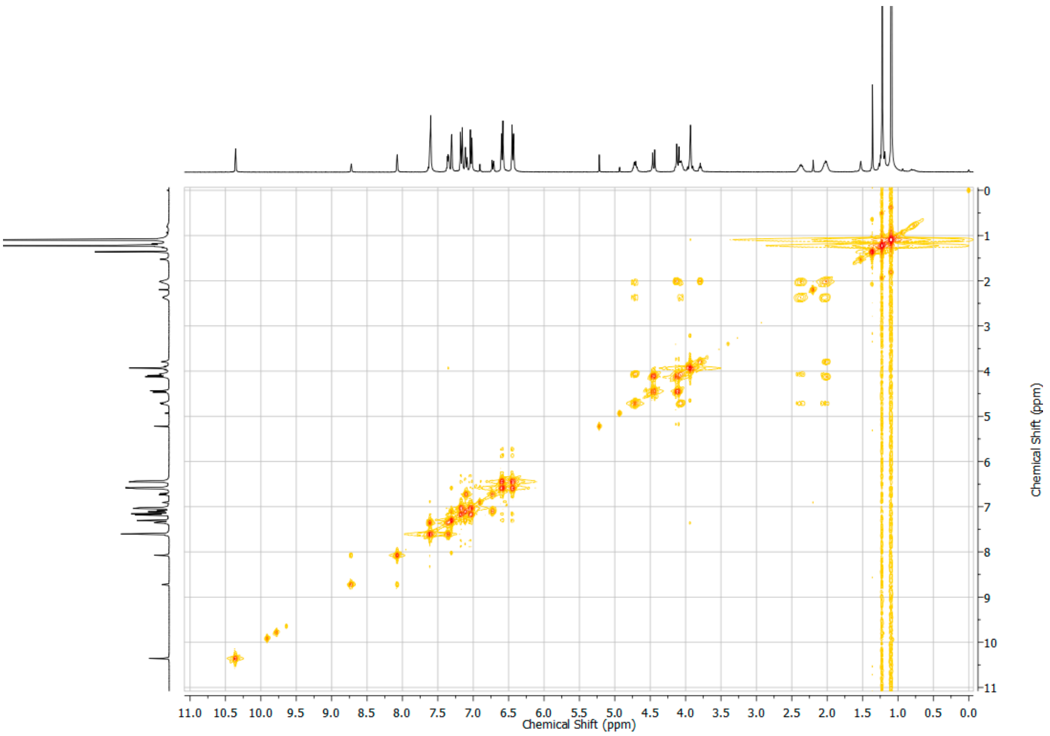

Figure S20. COSY NMR ($CDCl_3$) of **11b**.

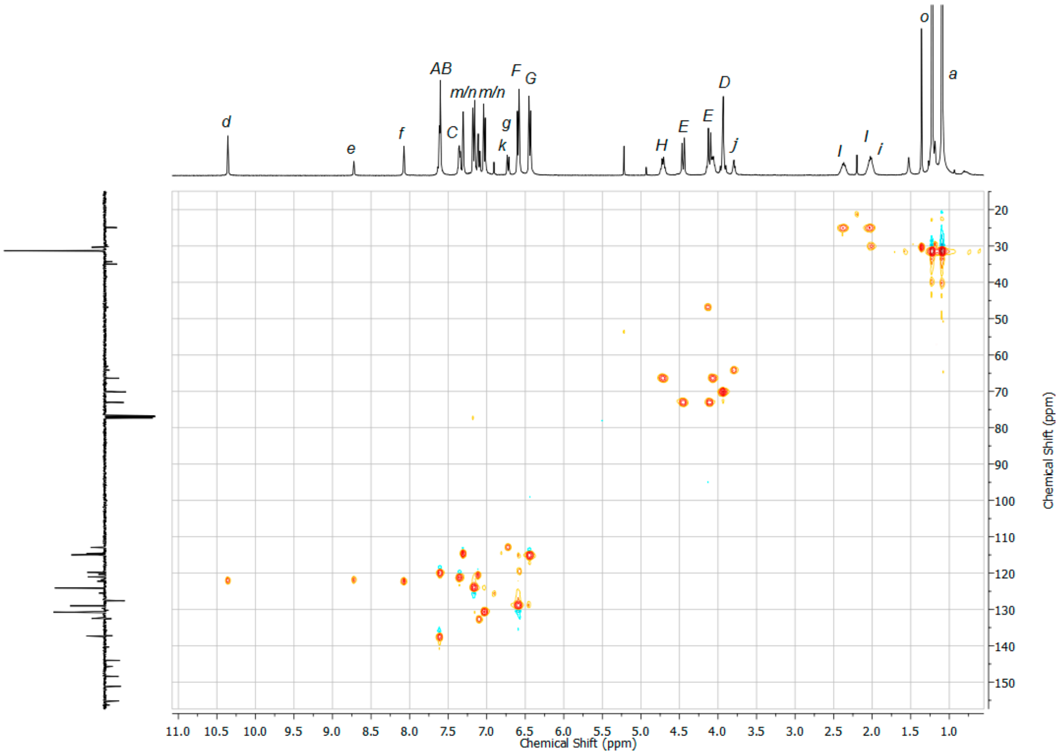

Figure S21. HSQC NMR ($CDCl_3$) of **11b**.

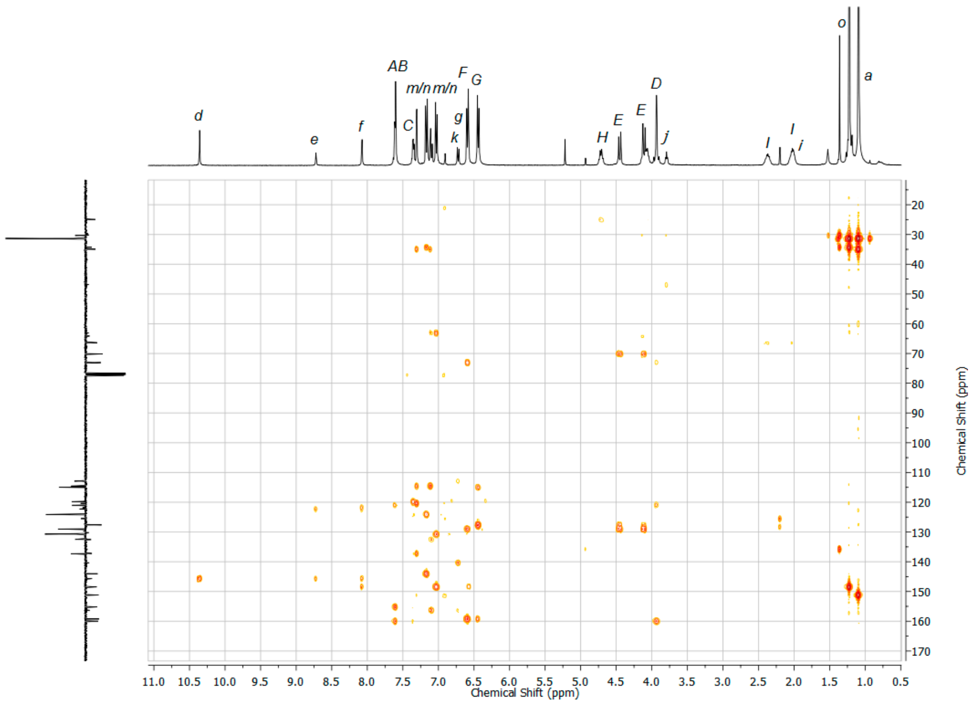

Figure S22. HMBC NMR ($CDCl_3$) of **11b**.

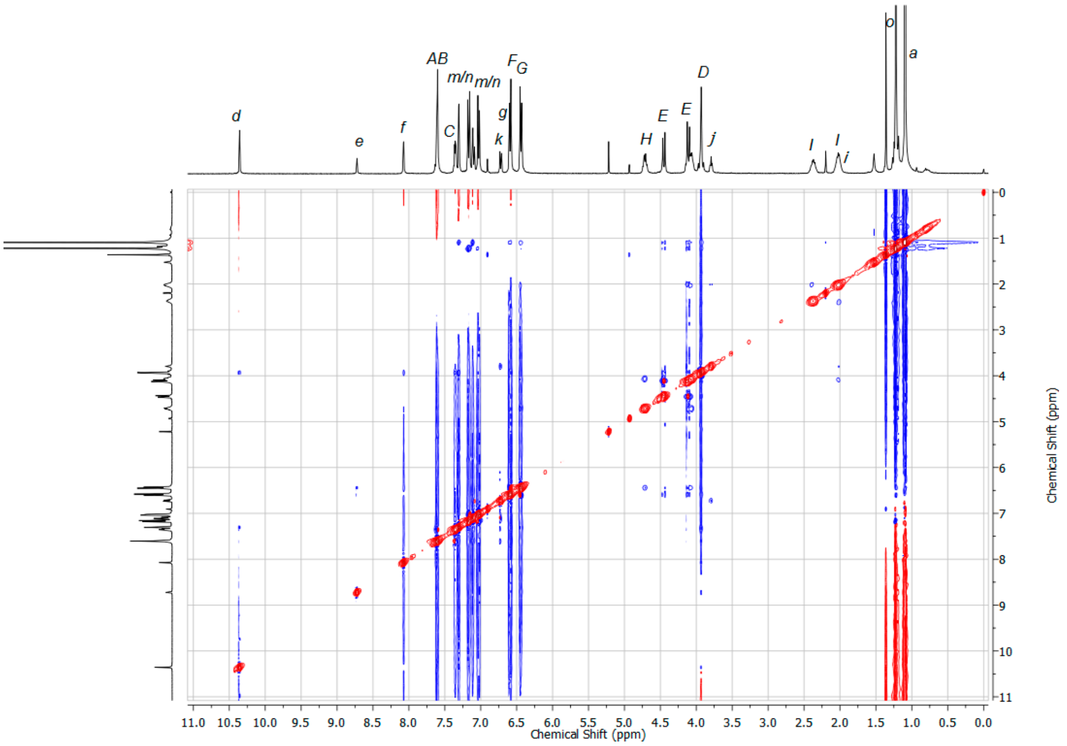

Figure S23. NOESY NMR ($CDCl_3$) of **11b**.

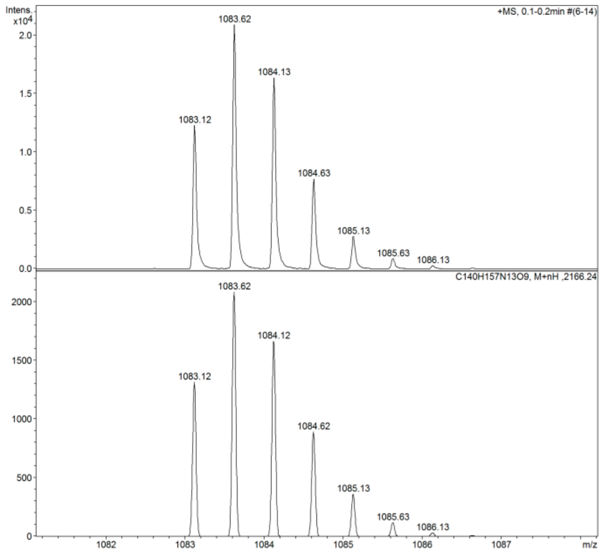

Figure S24. ESI-MS isotopic pattern of 11b; observed (**top**) and calculated (**bottom**).

[4]Rotaxane 12b

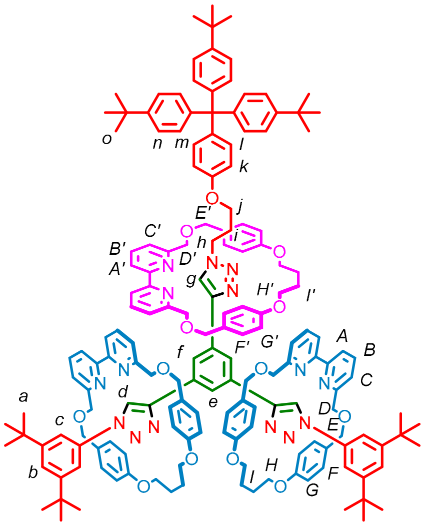

^{1}H-NMR ($CDCl_3$, 500 MHz, 298 K) δ: 10.56 (s, 2H, H_d), 8.79 (s, 1H, H_e), 8.22 (br. m, 2H, H_f), 7.65–7.59 (m, 6H, H_B, $H_{B'}$), 7.56–7.53 (m, 6H, H_A, $H_{A'}$), 7.47–7.56 (m, 2H, $H_{C'}$), 7.41 (s, 1H, H_g), 7.38 (d, J = 7.6, 4H, H_C), 7.32 (d, J = 1.7, 4H, H_c), 7.27–7.25 (m, 6H, H_n), 7.15 (s, 2H, H_b), 7.12 (d, J = 8.6, 6H, H_m), 6.87 (d, J = 8.6, 2H, $H_{k/l}$), 6.81 (d, J = 8.3, 4H, $H_{F'}$), 6.62 (d, J = 8.3, 8H, H_F), 6.51 (d, J = 8.4, 4H, $H_{G'}$), 6.42 (d, J = 8.3, 8H, H_G), 6.21 (d, J = 8.6, 2H, $H_{k/l}$), 4.71–4.67 (m, 4H, 4 of H_H), 4.60–4.52 (m, 6H, 4 of H_E, 2 of $H_{E'}$), 4.35–4.32 (m, 4H, 2 of $H_{D'}$, 2 of $H_{E'}$), 4.23 (d, J = 12.4, 2H, 2 of $H_{D'}$), 4.17–4.14 (6H, 4 of H_E, 2 of $H_{H'}$), 4.09 (d, J = 13.3, 4H, 4 of H_D), 4.00 (d, J = 13.3, 4H, 4 of H_D), 3.93 (br. m, 6H, 4 of H_H, 2 of $H_{H'}$), 3.30–3.25 (m, 2H, $H_{h/j}$), 2.89 (t, J = 6.9, 2H, $H_{h/j}$), 2.37 (br. m, 4H, 4 of H_I), 2.07–1.92 (m, 8H, 4 of H_I, $H_{I'}$), 1.32 (s, 27H, H_o), 1.13 (s, 36H, H_a), 0.84 (br. m, 2H, H_i). ^{13}C-NMR ($CDCl_3$, 126 MHz, 298 K) δ: 159.8, 159.0, 158.9, 156.6, 156.3, 155.3, 151.0, 148.2, 147.1, 145.7, 144.4, 138.6, 137.2, 137.1 (×2), 135.2, 132.8, 131.6, 131.3, 130.7, 130.2, 128.9, 128.8, 127.7, 124.0, 122.4, 122.1, 122.0, 121.1, 120.7, 120.5, 120.3, 119.9 (×2), 115.2, 114.9, 114.6, 113.1, 72.9, 72.8, 70.7, 70.2, 66.7, 66.1, 64.0, 63.1, 46.3, 34.9, 34.3, 31.4, 31.3, 29.0, 24.9, 24.8. ESI-MS m/z = 883.16 $[M + 3H]^{3+}$ calc. 883.16; 1324.23 $[M + 2H]^{2+}$ calc. 1324.23.

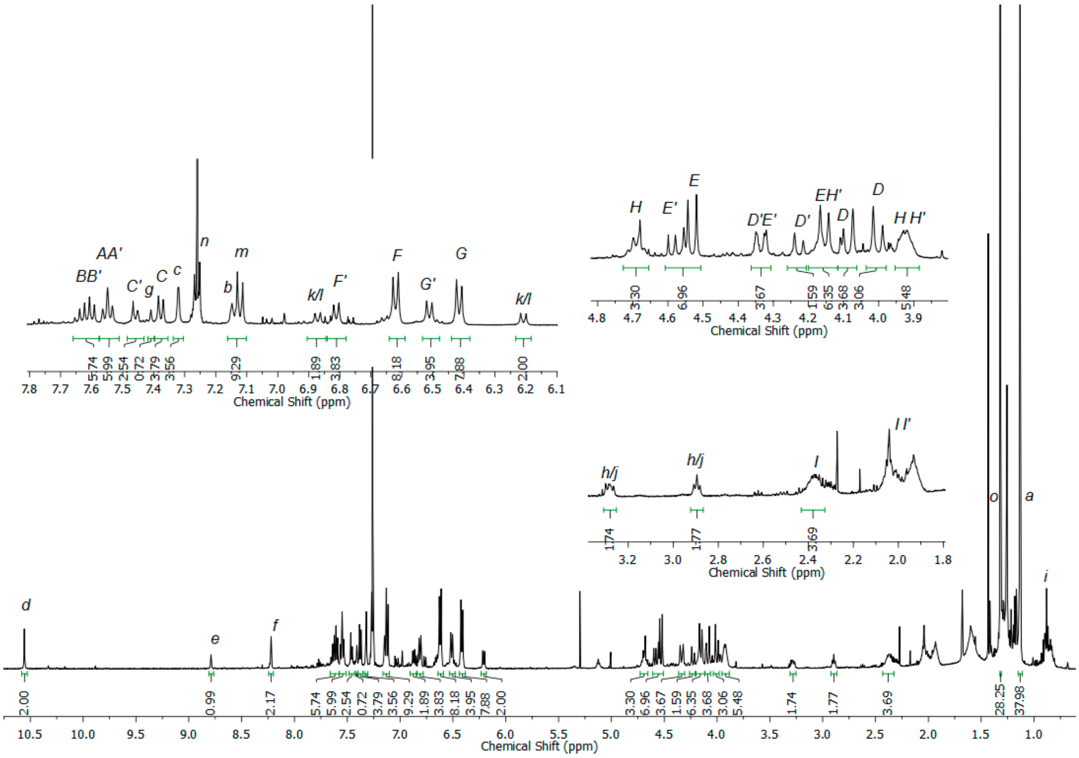

Figure S25. ^{1}H-NMR ($CDCl_3$, 500 MHz) of **12b**.

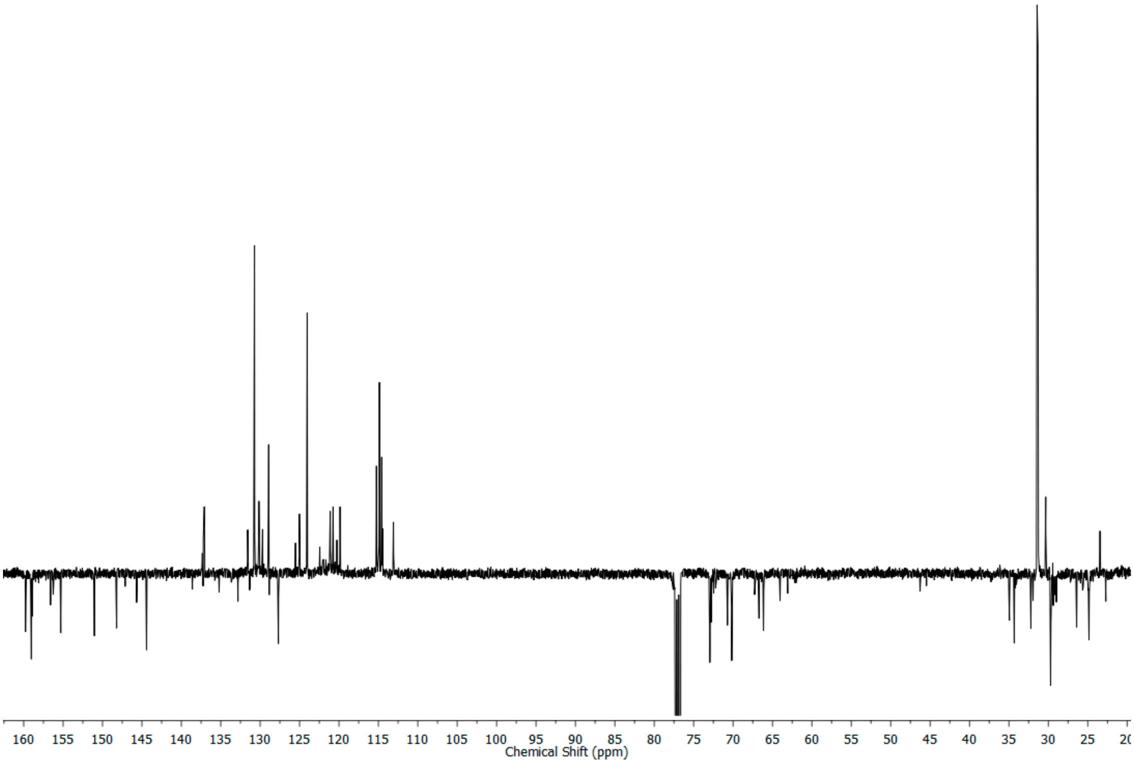

Figure S26. JMOD NMR ($CDCl_3$, 126 MHz) of **12b**.

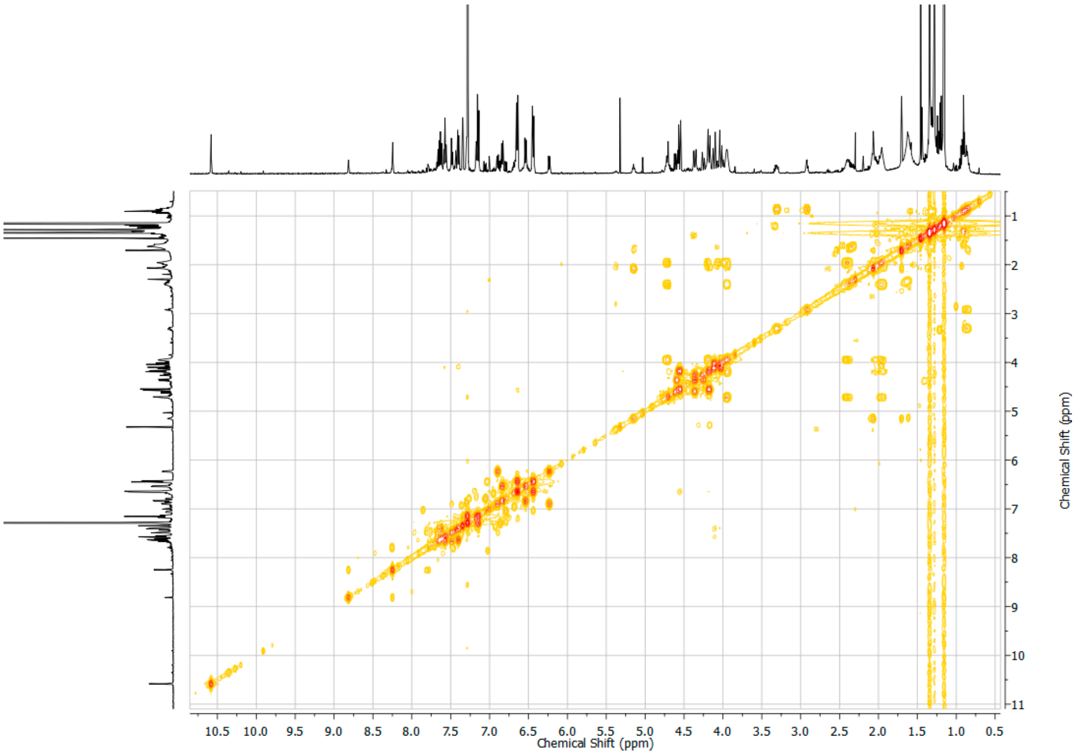

Figure S27. COSY NMR ($CDCl_3$) of **12b**.

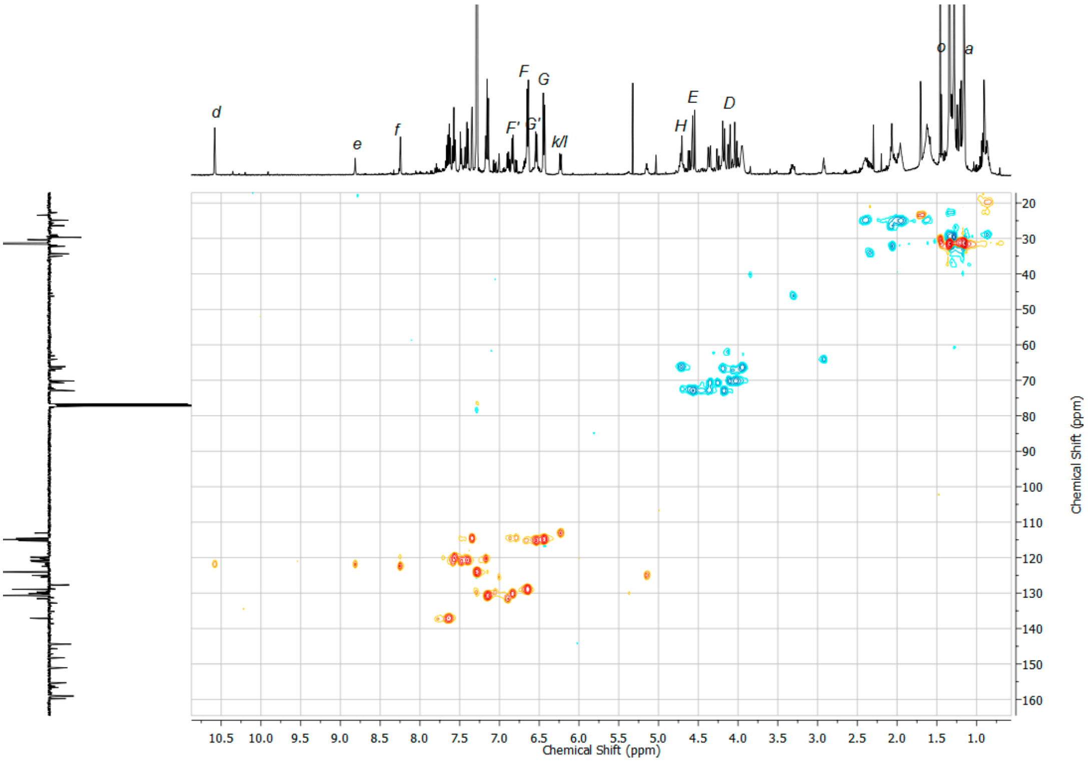

Figure S28. HSQC NMR ($CDCl_3$) of **12b**.

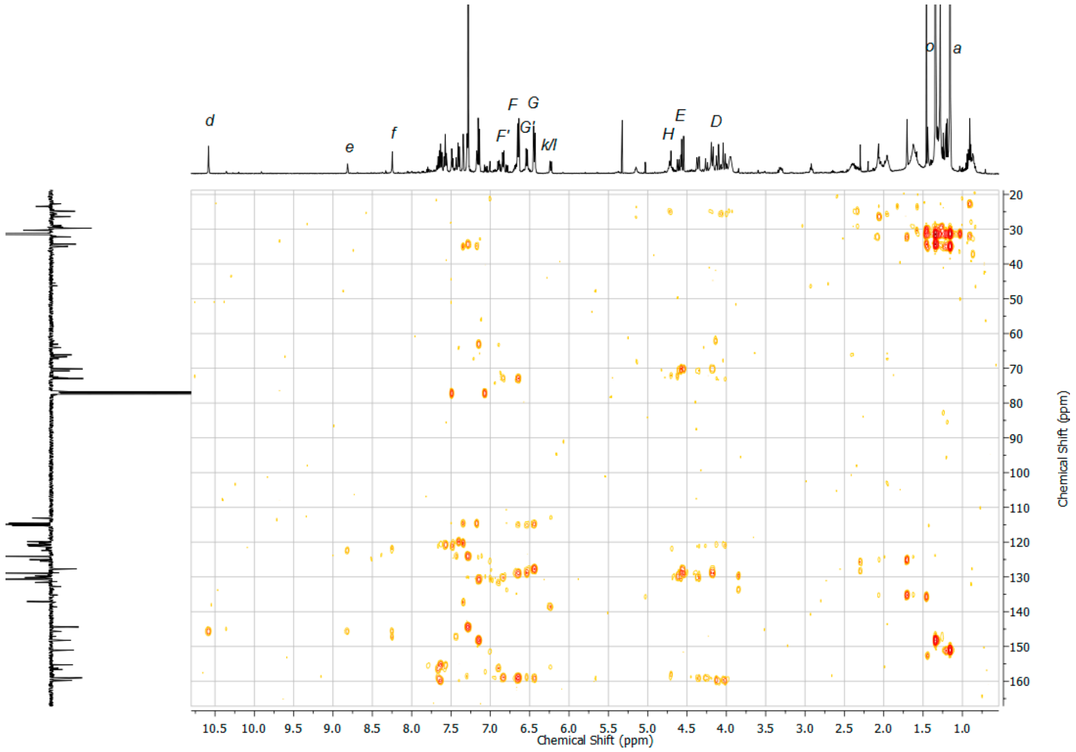

Figure S29. HMBC NMR (CDCl$_3$) of **12b**.

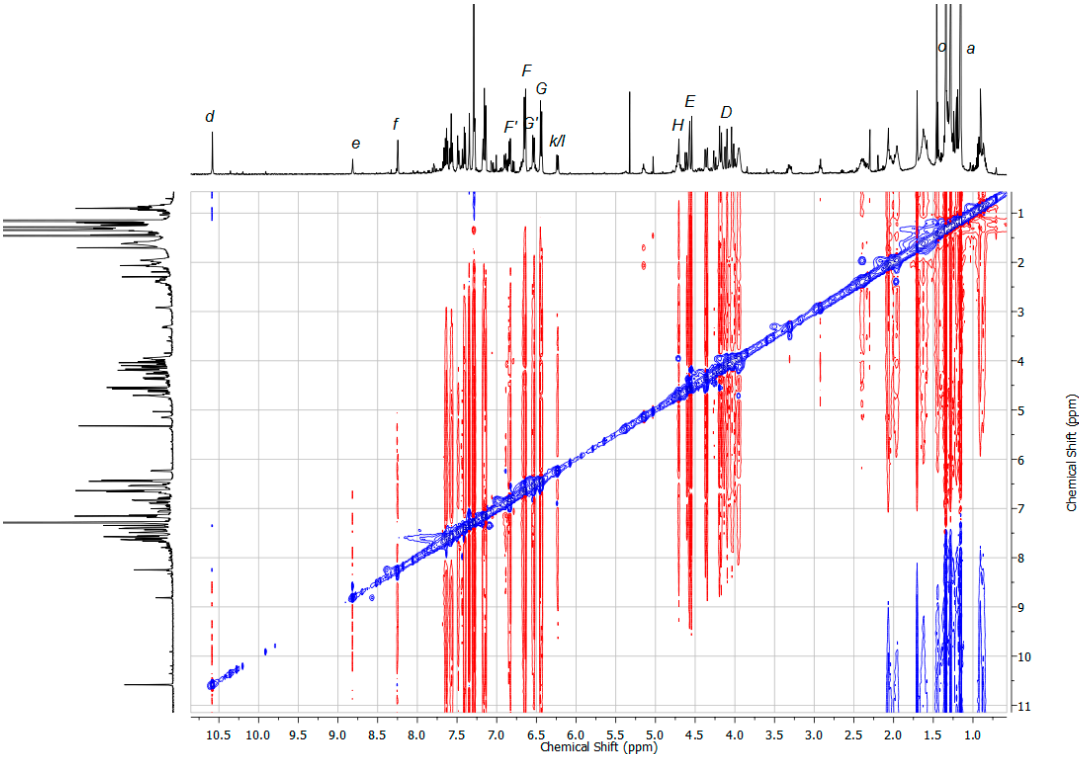

Figure S30. NOESY NMR (CDCl$_3$) of **12b**.

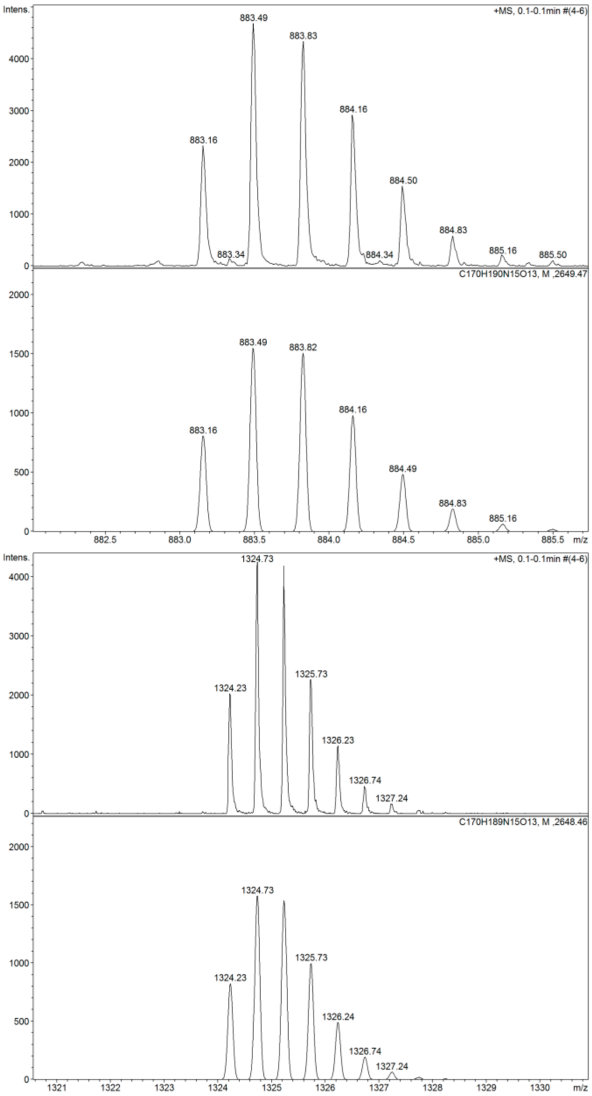

Figure S31. ESI-MS isotopic patterns of 12b; observed (**top**) and calculated (**bottom**).

General procedure. **8** (20.5 mg, 0.013 mmol, 1 eq.), **10** (7.6 mg, 0.013 mmol, 1 eq.), **9** (7.4 mg, 0.013 mmol, 1 eq.), [Cu(CH_3CN)$_4$](PF_6) (4.6 mg, 0.013 mmol, 1 eq.). Purification by column chromatography on silica gel (petrol with 0 to 100% gradient of Et_2O) yielded **11b** (17.9 mg, 63%) and **12c** (9.7 mg, 27%) as white foams.

[4]Rotaxane 12c

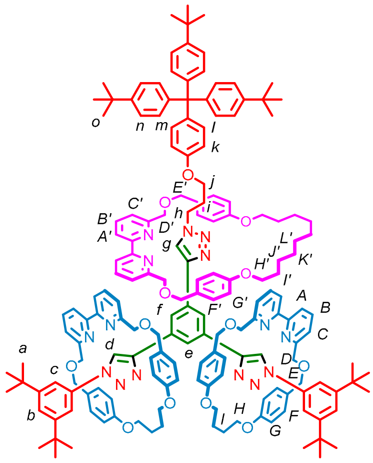

^{1}H-NMR ($CDCl_3$, 500 MHz, 298 K) δ: 10.50 (s, 2H, H_d), 8.79 (s, 1H, H_e), 8.13 (s, 2H, H_f), 8.00 (d, *J* = 7.7, 2H, $H_{A'}$), 7.68–7.65 (m, 8H, H_A, H_B), 7.45–7.42 (m, 6H, H_C, $H_{B'}$), 7.38 (s, 4H, H_c), 7.32 (d, *J* = 7.9, 2H, $H_{C'}$), 7.30 (d, *J* = 8.7, 6H, $H_{m/n}$), 7.18 (s, 2H, H_b), 7.13 (d, *J* = 8.7, 6H, $H_{m/n}$), 6.96 (d, *J* = 8.6, 4H, $H_{F'}$), 6.82 (d, *J* = 8.8, 2H, H_l), 6.65 (d, *J* = 8.5, 8H, H_F), 6.49–6.44 (m, 13H, H_G, $H_{G'}$, H_g), 5.94 (d, *J* = 8.7, 2H, H_k), 4.76 (br. m, 4H, 4 of H_H), 4.64–4.53 (m, 12H, 4 of H_E, $H_{D'}$, $H_{E'}$), 4.19 (d, *J* = 12.2, 4H, H_E), 4.08 (br. m, 4H, 4 of H_H), 4.04 (d, *J* = 13.3, 4H, 4 of H_D), 4.00 (d, *J* = 13.2, 4H, 4 of H_D), 3.73–3.67 (m, 4H, $H_{H'}$), 3.61 (br. m, 2H, $H_{h/j}$), 2.94 (br. m, 2H, $H_{h/j}$), 2.43 (br. m, 4H, 4 of H_I), 2.07 (br. m, 4H, 4 of H_I), 1.64–1.56 (m, 4H, $H_{I'}$), 1.34 (s, 27H, H_o), 1.34–1.10 (m, 12H, $H_{J'}$, $H_{K'}$, $H_{L'}$, H_i), 1.17 (s, 36H, H_a). ^{13}C-NMR ($CDCl_3$, 126 MHz, 298 K) δ: 160.1, 159.3, 158.7, 158.3, 156.1, 155.6, 155.3, 151.3, 148.4, 145.8, 144.5, 139.5, 137.5, 137.4, 137.3, 132.8, 131.9, 130.9, 130.6, 129.9, 129.8, 129.2, 127.8, 124.3, 122.3, 122.1, 121.9, 121.7, 121.1, 120.5, 120.0 (×2), 119.2, 115.1, 114.7, 114.4, 112.7, 73.2, 72.5, 72.4, 70.3, 67.6, 66.4, 63.9, 63.2, 47.0, 35.1, 34.5, 31.6, 31.5, 30.3, 29.6, 29.1, 29.0, 25.9, 25.0. ESI-MS *m*/*z* = 1366.27 [M + 2H]$^{2+}$ calc. 1366.28.

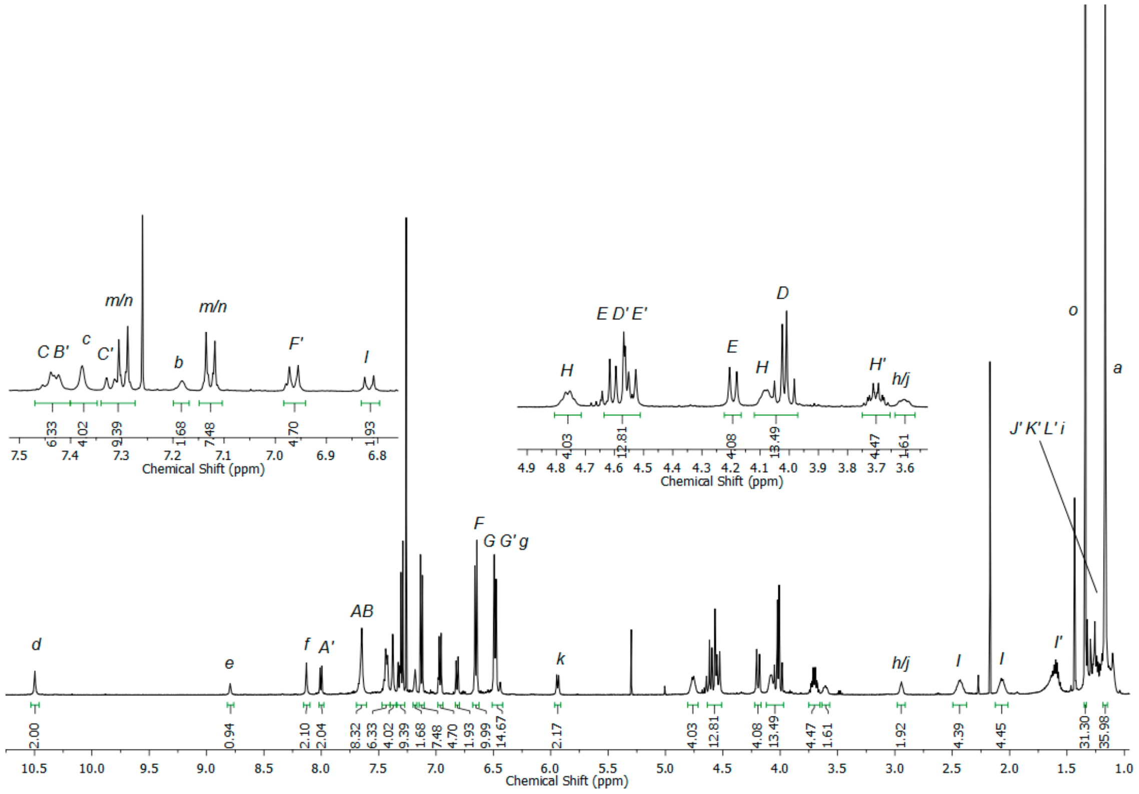

Figure S32. ^{1}H-NMR ($CDCl_3$, 500 MHz) of **12c**.

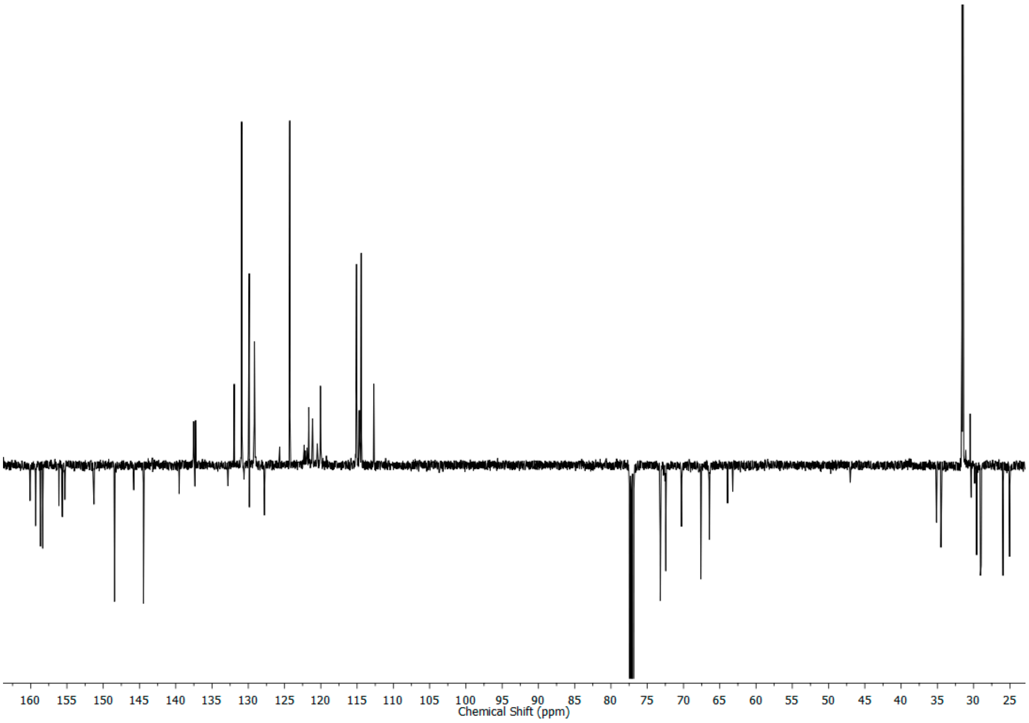

Figure S33. JMOD NMR ($CDCl_3$, 126 MHz) of **12c**.

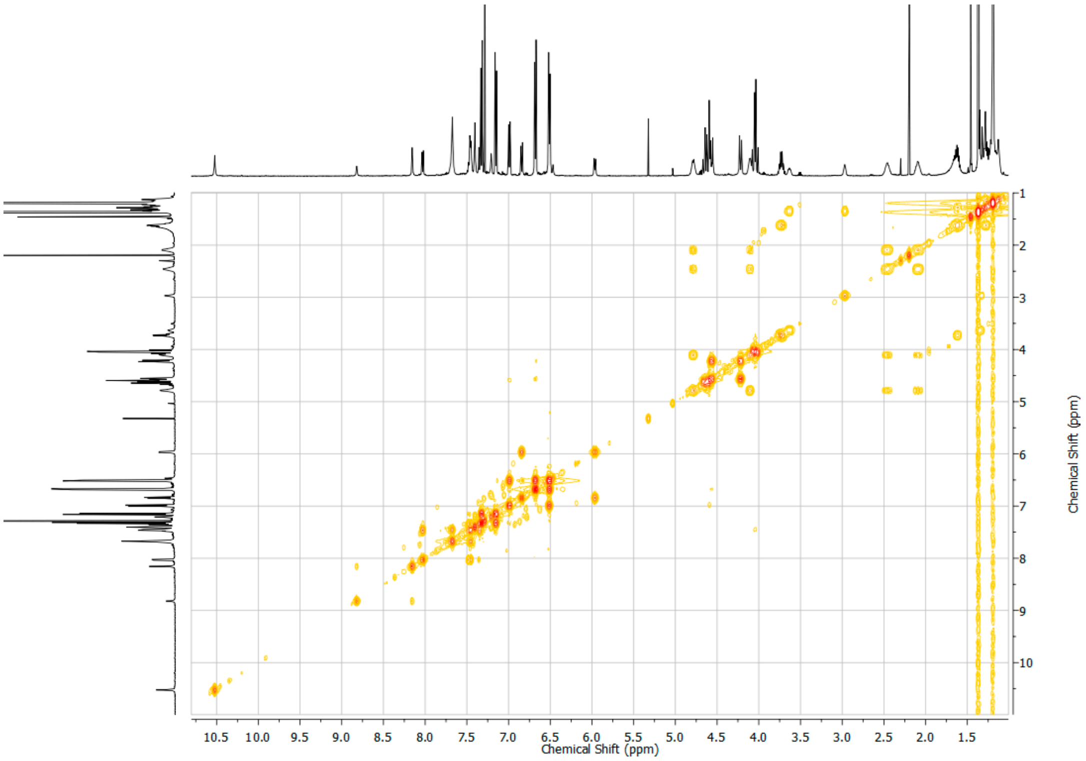

Figure S34. COSY NMR ($CDCl_3$) of **12c**.

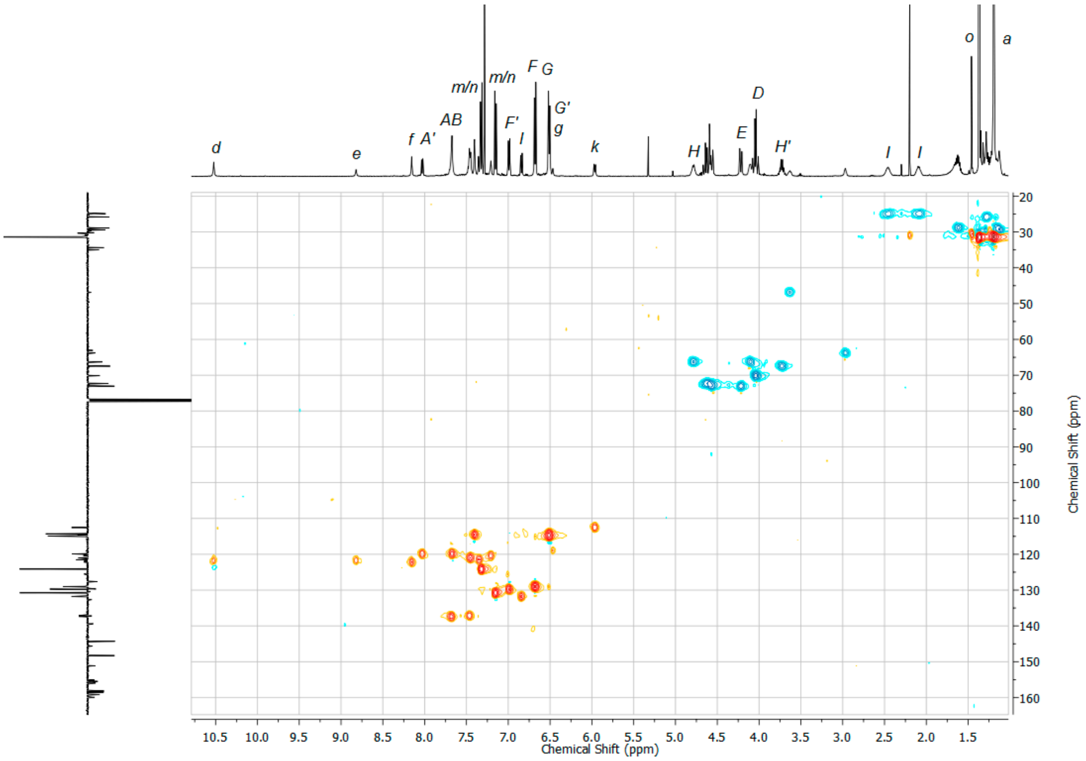

Figure S35. HSQC NMR ($CDCl_3$) of **12c**.

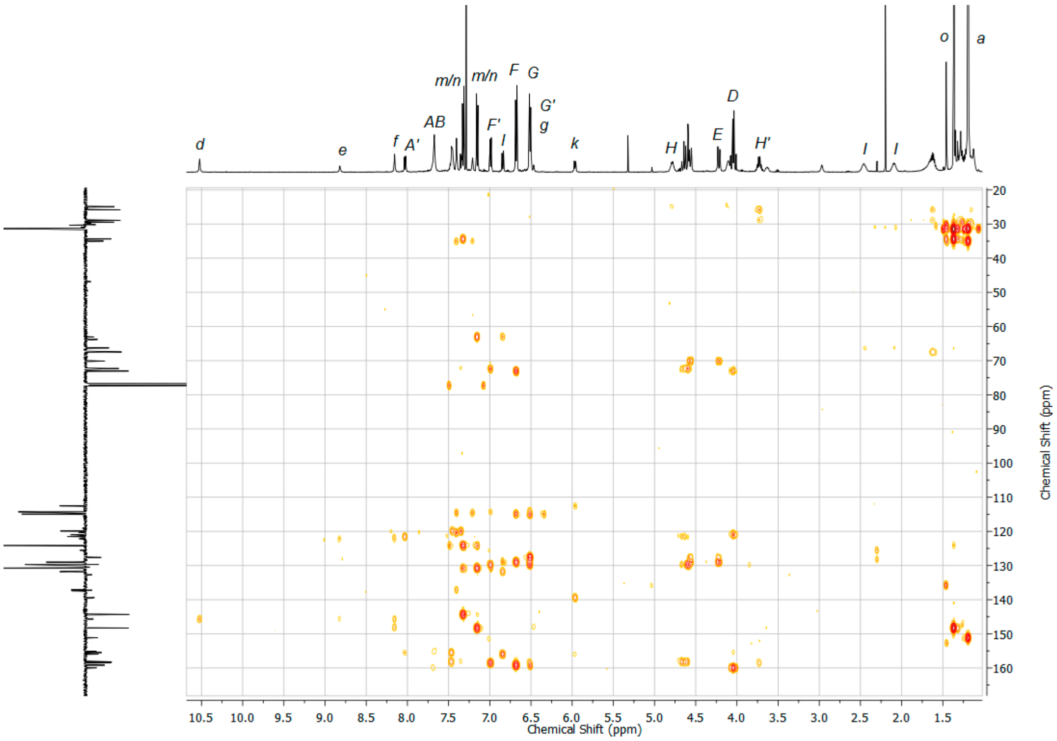

Figure S36. HMBC NMR ($CDCl_3$) of **12c**.

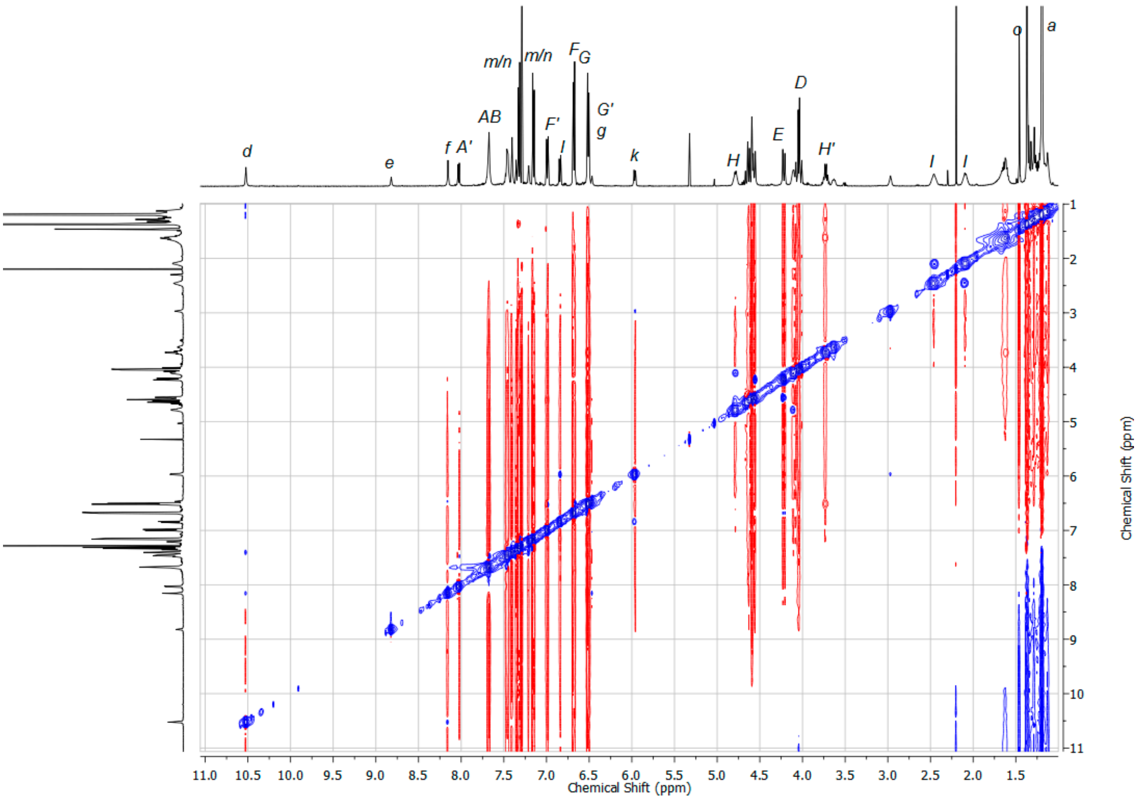

Figure S37. NOESY NMR (CDCl3) of **12c**.

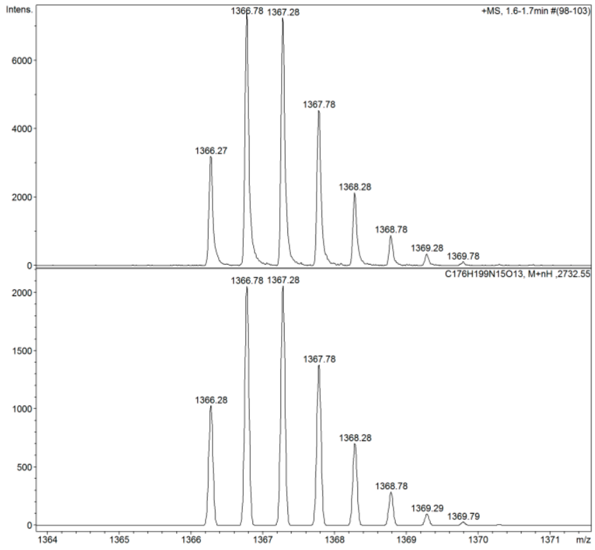

Figure S38. ESI-MS isotopic pattern of 12c; observed (**top**) and calculated (**bottom**).

Additional NMR and MS Data

Synthesis of [2]rotaxane 8

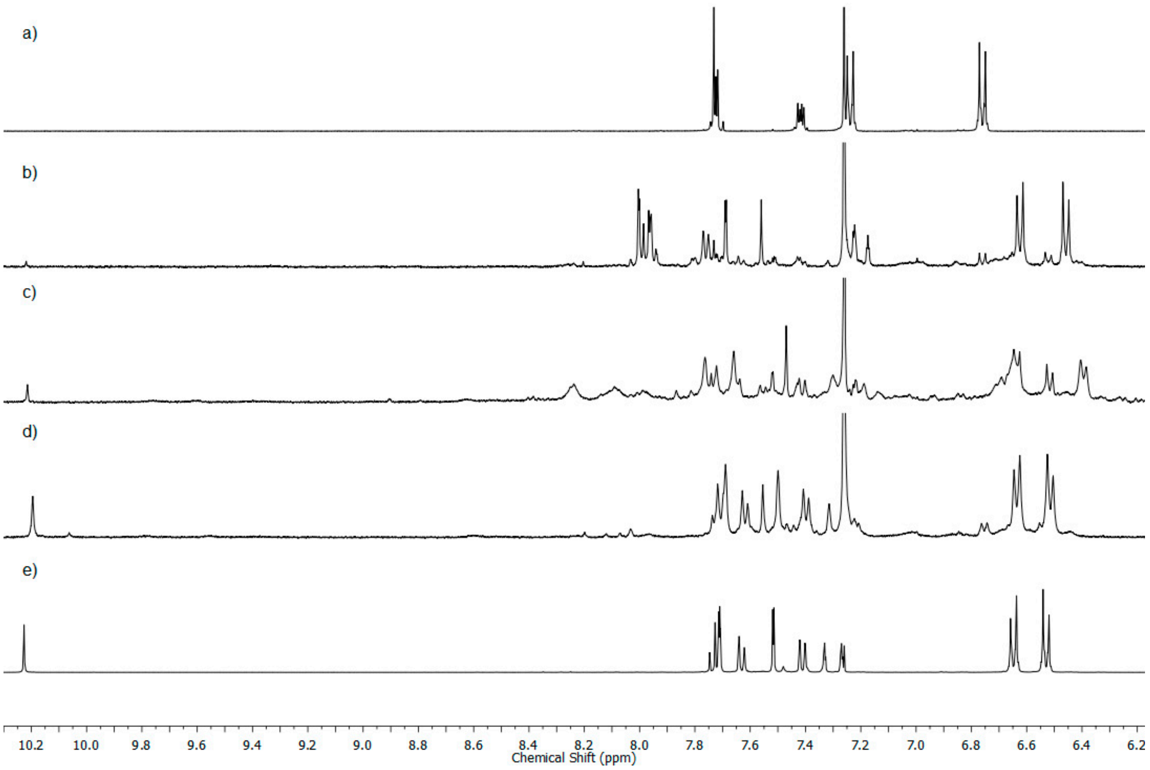

Figure S39. Partial ^{1}H-NMR (400 MHz, $CDCl_3$) of (**a**) macrocycle 3; (**b**) crude reaction mixture containing triazolide as major product; (**c**) crude reaction mixture following heating at 100 °C in CH_2Cl_2 for 1 h; (**d**) crude mixture after washing with EDTA-NH_3 solution, and (**e**) [2]rotaxane 8 after column chromatography.

Crude Reaction Mixtures

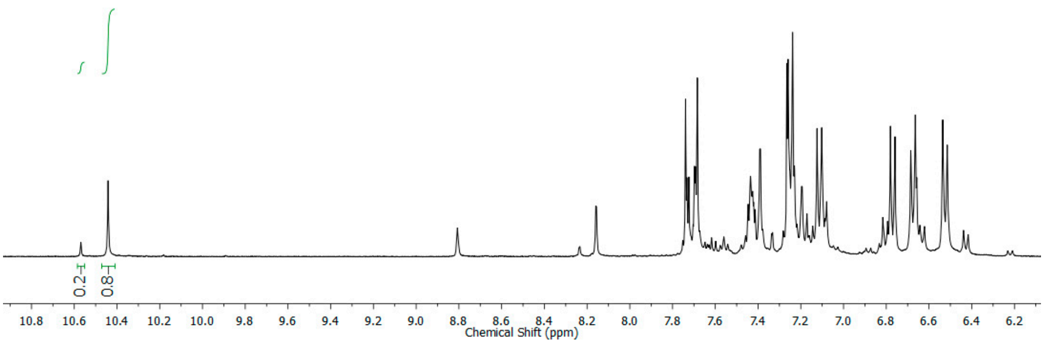

Figure S40. Partial ^{1}H-NMR (400 MHz, $CDCl_3$) of the reaction between [3]rotaxane 8, macrocycle 3 and azide 10.

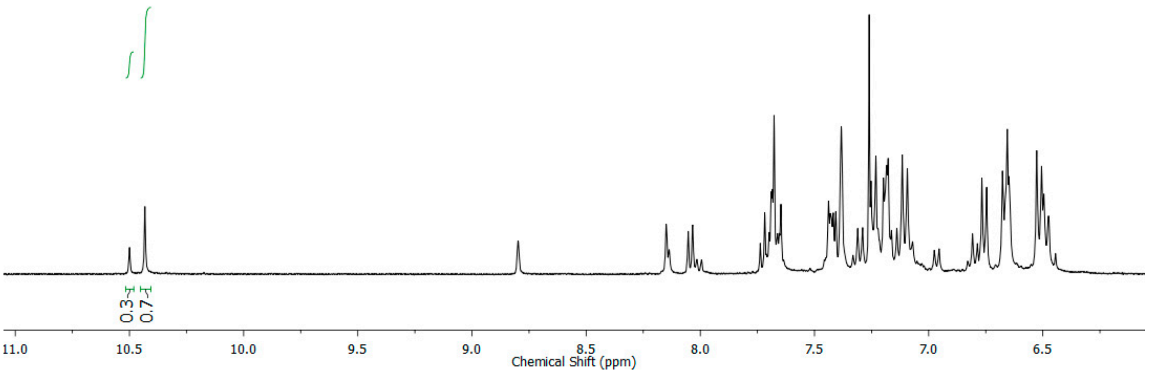

Figure S41. Partial ^{1}H-NMR (400 MHz, $CDCl_3$) of the reaction between [3]rotaxane 8, macrocycle 9 and azide 10.

MS Data

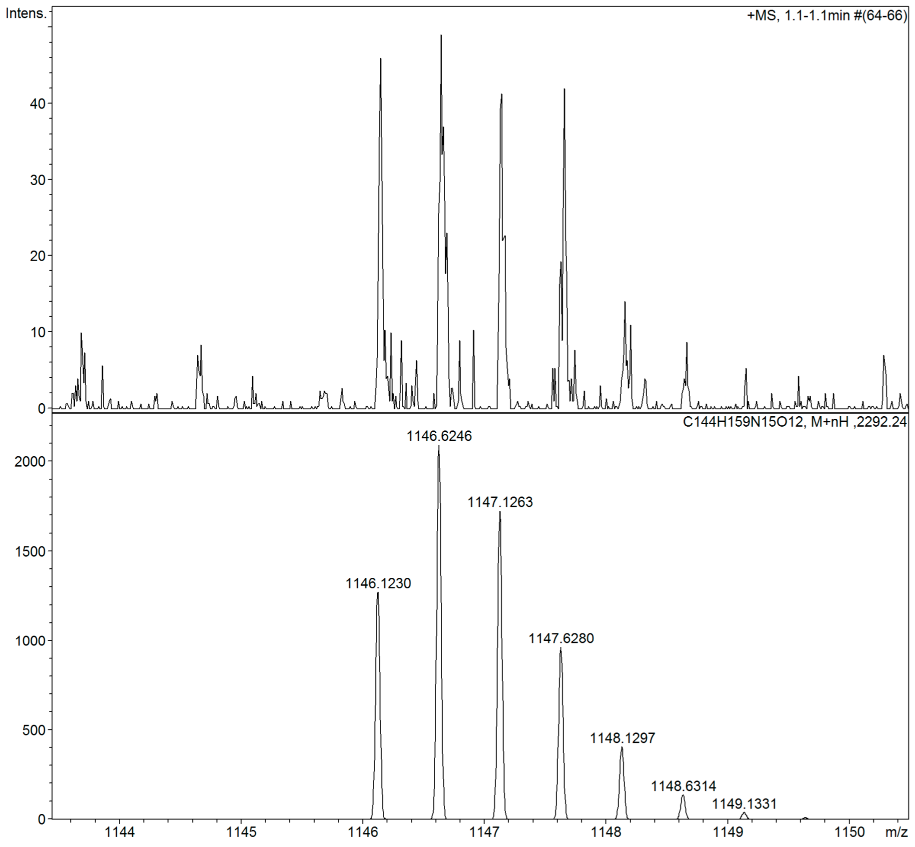

Figure S42. Observed (top) and calculated (bottom) $[M+2H]^{2+}$ isotope pattern for [4]rotaxane 12a.

X-ray Data for [3]Rotaxane 8

CCDC Number	1517622
Empirical formula	$C_{100}H_{108}N_{10}O_8$
Formula weight	1577.96
Temperature	100(2) K
Wavelength	0.71073 Å
Crystal system	Monoclinic
Space group	P 21/c
Unit cell dimensions	
a = 11.5354(2) Å	α= 90°
b = 20.9346(3) Å	β= 94.6970(10)°
c = 35.8102(5) Å	β = 90°

Volume	8618.7(2) Å^3
Z	4
Density (calculated)	1.216 Mg/m^3
Absorption coefficient	0.078 mm^{-1}
F(000)	3368
Crystal size	0.150 × 0.120 × 0.110 mm^3
Theta range for data collection	1.499 to 32.088°.
Index ranges	−16 ≤ h ≤13, −30 ≤ k ≤ 30, −52 ≤ l ≤ 52
Reflections collected	119054
Independent reflections	28,288 [R(int) = 0.0516]
Completeness to theta = 25.242°	100.0%
Absorption correction	Semi-empirical from equivalents
Max. and min. transmission	1.000 and 0.78239
Refinement method	Full-matrix least-squares on F^2
Data/restraints/parameters	28,288/0 1075
Goodness-of-fit on F^2	1.028
Final R indices [I>2sigma(I)]	R_1 = 0.0641, wR_2 = 0.1521
R indices (all data)	R_1 = 0.1074, wR_2 = 0.1752
Extinction coefficient	n/a
Largest diff. peak and hole	0.673 and −0.328 e.Å^{-3}

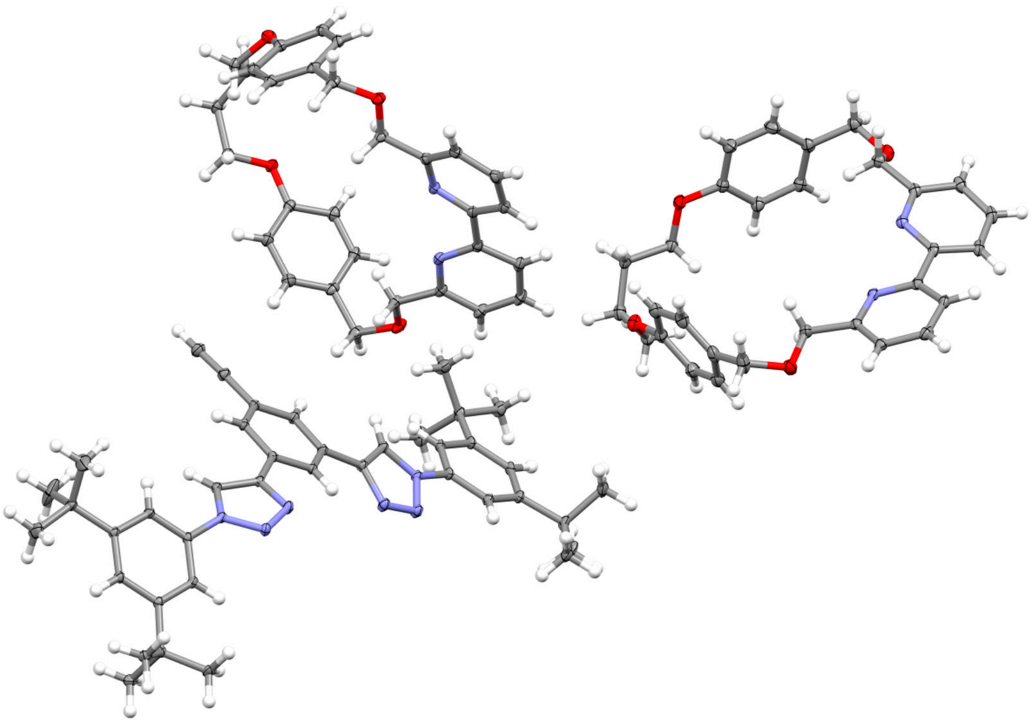

Figure S43. Ellipsoid plot of the asymmetric unit of 8. Ellipsoids are shown at the 50% probability level.

Crystals were grown by vapour diffusion of pentane into a CH_2Cl_2 solution. Data were collected at 100 K using a Rigaku 007 HF diffractometer equipped with a Saturn 944+ enhanced sensitivity detector. Cell determination and data collection were done using CrystalClear-SM Expert 3.1; data reduction, cell refinement and absorption correction were performed with CrysalisPro. The structure was solved using SUPERFLIP and refined against F2 using anisotropic thermal displacement parameters for all non-hydrogen atoms using WINGX and software packages within. Hydrogen atoms were placed in calculated positions and refined using a riding model.

References

1 Zhu, W.; Ma, D. Synthesis of aryl azides and vinyl azides via proline-promoted CuI-catalyzed coupling reactions. *Chem. Commun*. **2004**, 888–889, doi:10.1039/B400878B.

2 Kobayashi, N.; Kijima, M. 1,3,5-Tris(functionalised-phenylethynyl)benzene–metal complexes: synthetic survey of mesoporous coordination polymers and investigation of their carbonisation. *J. Mater. Chem.* **2008**, *18*, 1037–1045.

3 Aucagne, V.; Hänni, K.D.; Leigh, D.A.; Lusby, P.J.; Walker, D.B. Catalytic "Click" Rotaxanes: A Substoichiometric Metal-Template Pathway to Mechanically Interlocked Architectures. *J. Am. Chem. Soc*. **2006**, *128*, 2186.

4 Lewis, J.E.M.; Bordoli, R.J.; Denis, M.; Fletcher, C.J.; Galli, M.; Neal, E.A.; Rochette, E.M.; Goldup, S.M. High yielding synthesis of 2,2′-bipyridine macrocycles, versatile intermediates in the synthesis of rotaxanes. *Chem. Sci*. **2016**, *7*, 3154–3161.
